# A novel group parenting intervention to reduce emotional and behavioural difficulties in young autistic children: protocol for the Autism Spectrum Treatment and Resilience pilot randomised controlled trial

**DOI:** 10.1136/bmjopen-2019-029959

**Published:** 2019-06-27

**Authors:** Melanie Palmer, Joanne Tarver, Juan Paris Perez, Thomas Cawthorne, Renee Romeo, Dominic Stringer, Victoria Hallett, Joanne Mueller, Lauren Breese, Megan Hollett, Bryony Beresford, Martin Knapp, Vicky Slonims, Andrew Pickles, Emily Simonoff, Stephen Scott, Tony Charman

**Affiliations:** 1 Department of Child and Adolescent Psychiatry, King’s College London, Institute of Psychiatry, Psychology & Neuroscience, London, UK; 2 Department of Psychology, Aston University, School of Life and Health Sciences, Birmingham, UK; 3 Department of Health Service and Population Research, King’s College London, Institute of Psychiatry, Psychology & Neuroscience, London, UK; 4 Department of Biostatistics and Health Informatics, King’s College London, Institute of Psychiatry, Psychology & Neuroscience, London, UK; 5 Service for Complex Autism & Associated Neurodevelopmental Disorders, South London and Maudsley NHS Foundation Trust, London, UK; 6 Social Policy Research Unit, University of York, York, UK; 7 Personal Social Services Research Unit, London School of Economics, London, UK; 8 Evelina Children’s Hospital, Guy’s and St Thomas' NHS Foundation Trust, London, UK; 9 Adoption and Fostering Service & Conduct Problems Service, South London and Maudsley NHS Foundation Trust, London, UK; 10 Department of Psychology, King’s College London, Institute of Psychiatry, Psychology & Neuroscience, London, UK

**Keywords:** Autism, emotional and behavioural difficulties, parenting intervention, feasibility, pilot RCT

## Abstract

**Introduction:**

The majority of young autistic children display impairing emotional and behavioural difficulties that contribute to family stress. There is some evidence that behavioural parenting interventions are effective for reducing behavioural difficulties in autistic children, with less evidence assessing change in emotional difficulties. Previous trials have tended to use unblinded parent-report measures as primary outcomes and many do not employ an active control, limiting the conclusions that can be drawn.

**Methods and analysis:**

The Autism Spectrum Treatment and Resilience study is a pilot randomised controlled trial (RCT) testing the specific effect of a 12-week group parenting intervention (Predictive Parenting) on primary and secondary outcomes, in comparison to an attention control condition consisting of psychoeducation parent groups. Following a feasibility study to test research procedures and the interventions, the pilot RCT participants include 60 parents of autistic children aged 4–8 years who are randomised to Predictive Parenting versus the attention control. Measures are administered at baseline and post intervention to assess group differences in child and parent outcomes, costs and service use and adverse events. The primary outcome is an objective measure of child behaviours that challenge during interactions with their parent and a researcher. The trial aims to provide data on recruitment, retention, completion of measures and acceptability of the intervention and research protocol, in addition to providing a preliminary indication of potential efficacy and establishing an effect size that could be used to power a larger-scale efficacy trial. We will also provide preliminary estimates of the cost-effectiveness of the interventions.

**Ethics and dissemination:**

Ethical approval was granted from NHS Camden and Kings Cross Research Ethics Committee (ref: 16/LO/1769) along with NHS R&D approval from South London and Maudsley, Guy’s and St Thomas', and Croydon Health Services NHS Trusts. The findings will be disseminated through publication in peer-reviewed journals and presentations at conferences.

**Trial registration number:**

ISRCTN91411078

**Strengths and limitations of this study**

- The trial uses an objective measure as the primary outcome overcoming biases associated with participants being unblinded to treatment status.
- The target intervention, developed by clinicians with expertise in autism, is compared to an attention control condition to further guard against placebo effects.
- A feasibility study with nested qualitative evaluation enabled refinement of the intervention and research procedures prior to commencing the pilot randomised controlled trial (RCT).
- Parents and autistic adults, referred to as patient and public involvement panels, were involved in the development of the interventions and research procedures.
- As the study is a pilot RCT, conclusions about the efficacy of the intervention are not possible.

## Introduction

### Background

Autism is characterised by difficulties in reciprocal social communication and the presence of restricted interests, repetitive behaviours and sensory anomalies.^1^ At least 1% of children are autistic,^2–4^ and the condition is around three to four times more prevalent in males than in females.^5^ There are high rates of intellectual disability in autistic children with approximately 55% having an IQ <70.^6^ It has been demonstrated that additional psychiatric disorders frequently co-occur with autism at rates much higher than in the general population; up to 80%–90% of young autistic children have additional emotional or behavioural difficulties meeting formal diagnostic criteria, with many having two or more additional disorders.^7–9^ Anxiety disorders, attention deficit/hyperactivity disorder and opposition defiant disorder are most common, and these difficulties tend to persist over time.^10^

Parents often report that it is these co-occurring difficulties, which are associated with poorer parental well-being and parental stress,^11^ that they would like support with. Universal interventions are warranted given the high prevalence of co-occurring emotional and behavioural difficulties in autistic children. However, current service provision in the UK usually includes the offer of psychoeducation groups that focus on teaching parents about autism and developing strategies to support social and communication functioning, rather than the commonly co-occurring emotional and behavioural difficulties.

Behavioural parenting interventions are recommended by the National Institute of Health and Care Excellence^12^ for the treatment of behavioural difficulties displayed by young children without autism. There are a number of effective parenting interventions that aim to reduce such difficulties in young autistic children. A recent meta-analysis of eight randomised controlled trials (RCTs) of behavioural parenting interventions aiming to reduce disruptive behaviour displayed by young autistic children^13^ found a moderate effect on disruptive behaviour when compared with controls (standardised mean difference=−0.59, 95% CI −0.88,–0.30). However, there was significant heterogeneity in the effect of parenting interventions on disruptive behaviour which may be due to sample size, mode of delivery and the focus and duration of treatment. Only one RCT included in the review included anxiety management techniques even though anxiety disorders are the most common co-occurring psychiatric diagnoses in autism, and ‘behaviours that challenge’ are often described as an observable manifestation of anxiety.^14 15^ A recent meta-analysis of 14 RCTs of cognitive behavioural therapy interventions for anxiety in young autistic children, most of which included parental components, demonstrated that reductions in anxiety could be achieved.^16^

In addition, only one parenting intervention reviewed by Postorino *et al* 13 included group-based sessions for parents, even though groups are more scalable and have the added benefit of providing a support network for parents. More than half of the included RCTs compared parenting interventions to a waitlist control or care as usual,^13^ limiting conclusions that can be drawn about the effects as participants would not be blinded to treatment allocation. Being unblinded to treatment allocation is particularly problematic when self-report measures are used as primary outcomes.^17^ There is a need for objective blinded measures of behaviour to be used as outcome measures in trials aiming to reduce emotional and behavioural difficulties displayed by young autistic children.

### Aims and objectives

The Autism Spectrum Treatment and Resilience (ASTAR) trial is part of a research programme that aims to improve mental health outcomes among autistic individuals (Improving Autism Mental Health: https://iamhealthkcl.net/). ASTAR tests the specific effect of the Predictive Parenting intervention on child emotional and behavioural difficulties, in comparison to an attention control condition (psychoeducation parent groups). The aims of the ASTAR trial are to 1) examine the feasibility of the intervention in terms of recruitment, retention, completion of research measures and acceptability to parents; 2) provide a preliminary indication of potential efficacy on the primary and secondary outcomes and establish an effect size (ES) that could be used to power a future larger-scale RCT; and 3) provide preliminary estimates of the cost-effectiveness of the intervention to inform a larger trial.

Consistent with Medical Research Council guidance on evaluating complex interventions,^18^ we first conducted a preliminary feasibility phase testing the proposed research procedures and the Predictive Parenting (target intervention) and psychoeducation (control) group interventions with families with an autistic child aged 4–8 years. A nested qualitative evaluation was conducted to explore the views of parents who declined to take part, those who completed/dropped out of the interventions and the group facilitators. Findings from the feasibility phase were used to amend the research procedures and intervention manuals prior to the subsequent pilot RCT (see below for further information on learning from the feasibility phase).

The primary outcome of the pilot RCT is observed child behaviours that challenge, captured during a structured researcher–child and parent–child interaction assessment (see description of measure below for further details). Secondary outcomes are child compliance and child-centered and child-directive parenting captured from the same observation and parent and teacher report of child emotional and behavioural difficulties. We are also measuring the effects of the interventions on parental stress and well-being, parenting practices and parenting self-efficacy.

## Methods and analysis

### Learning from the feasibility phase

The aim of the feasibility phase was to test the proposed recruitment processes and rates, the adequacy and acceptability of proposed measures and obtain the views of parents and professionals on the research processes and interventions. Participants were 22 families (91% mothers and 9% fathers) with a child aged 4–8 years with a clinical diagnosis of autism spectrum disorder (ASD). All but one of the children were male, and were spilt across mainstream (n=10) and two special schools (n=12). Children in the special schools groups attended either a mixed autism-specific special school or a special school catering for children with severe learning difficulties co-occurring with autism. As intervention content is differentiated by child verbal ability, parents of minimally verbal children (n=12) attended groups separately from parents of verbal children (n=10).

We recruited 22 out of our target of 24 (92%) for the feasibility phase and we retained 20/22 (91%) families in the research protocol to post intervention, indicating that the research processes were acceptable to families. All 22 parents gave consent for their child’s teacher to complete measures. Baseline teacher questionnaires were obtained for 20/22 (91%) children and retention of teachers at post intervention was high (18/22, 82%).

Parents who were interviewed reported that the research procedures were acceptable, although some felt the assessment process was lengthy. Prior to commencing the pilot RCT, two proposed outcome measures were removed to reduce burden on families (see our ISRCTN record for a log of outcome measures tested during the feasibility phase). For some parents, there appeared to have been a lack of clarity about the difference between the research and clinical teams and who they would have contact with at each stage of the study. This led to amendments in the information given to parents to help make this distinction clearer. Findings from the qualitative interviews also indicated that most parents reported that they found the groups helpful and that they enjoyed meeting other parents in a similar situation. Feedback on the structure, timing, course materials and homework led to modifications to the Predictive Parenting intervention. For example, changes were made to make the groups more accessible and relevant to parents of children with lower levels of verbal ability. The study design was also amended by increasing the number of families in each group (from six to eight) as it was a more efficient way to recruit and deliver the interventions. The increased group size was not thought to disrupt the intervention; indeed, the slightly larger sizes may be helpful for group dynamics. Further details on the feasibility study can be provided on contact with the research team.

### Patient and public involvement

Panels of parents of autistic children and autistic adults have been involved in all phases of the study and assisted with the development of the intervention curriculums and adaptations for parents of minimally verbal children, as well as advising on the research procedures. Guidance and advice about language to use when speaking with parents about the therapy goals and research processes (including on the written materials such as flyers and information sheets) was given.

### Trial design

The study is a parallel-group pilot RCT. Participating families are allocated to one of two treatment arms (Predictive Parenting or psychoeducational parent groups). Randomisation is conducted on blocks of 10–18 families on a ratio of 1:1, resulting in groups of 5–9 families in each treatment arm for any block. The randomisation algorithm is run by an independent statistician within the Biostatistics and Health Informatics Department, IoPPN, King’s College London. Details of this are recorded in a separate randomisation specification document. Intervention allocation is emailed only to the group facilitators to ensure that the researchers are blinded to condition.

Measures are collected at baseline, up to 2 months prior to the planned randomisation date and approximately 18–24 weeks after randomisation once the 12-week intervention has finished. Group differences in outcomes will be examined.

### Inclusion criteria

- Parent/carer of an autistic child, as confirmed by their clinician, aged between 4:0 years and 8:11 years.
- Have sufficient spoken English to access the intervention.
- Agree that their family doctor can be informed of their involvement in the trial.

### Exclusion criteria

- Current participation in a behavioural parenting intervention delivered by another service.
- Child has epileptic seizures more than weekly.
- Parent or child has a severe hearing or visual impairment.
- Active significant safeguarding concerns or a current severe parental psychiatric disorder.
- Participation in the initial feasibility phase.

### Interventions

#### Predictive Parenting (target intervention)

Predictive Parenting builds on behavioural parenting interventions, an evidence-based, well-accepted and cost-effective approach to targeting disruptive behaviour in children without autism.^12^ It also incorporates well-established parent-mediated cognitive-behavioural therapy strategies for managing child anxiety.^16^ It consists of 12 weekly 2-hour groups, which extend parents’ understanding of autism and associated difficulties and focus on supporting parents to understand and manage their child’s emotions and behaviours (see table 1 for content covered in Predictive Parenting). Techniques for helping parents prevent and reduce disruptive behaviour and anxiety are taught. It also includes content on promoting parental self-care and stress reduction. Content is adapted based on child verbal ability (minimally verbal vs verbal). In addition to the 12 group sessions, two individual sessions are conducted—one between sessions 2 and 4 and the other between sessions 10 and 12. These individual sessions are up to 60 min long and aim to support individualisation and generalisation of the strategies for each family. The intervention is conducted in the community in local child and adolescent mental health services, libraries or schools. Further information about Predictive Parenting will be published in a separate manuscript.

**Table 1:** Content covered in Predictive Parenting

Group session	Content
1	Understanding ASD
2	Becoming a behaviour predictor
3	The power of planning
4	Predictably positive household
5	Clever communication
6	Predictable praise and rewards
7	Managing challenging behaviour and meltdowns
8	Predictable parent action plans
9	Understanding anxiety
10	Anxiety and Unpredictability Toolkit 1
11	Anxiety and Unpredictability Toolkit 2
12	Looking forward and looking after yourself

ASD, autism spectrum disorder.

### Psychoeducational parent group (attention control condition)

‘The Seven Cs of ASD’, the attention control condition, also consists of 12 weekly 2-hour groups that aim to provide psychoeducation and social support, while not providing specific guidance on managing behaviours or emotions. Table 2 displays the content covered in each session of The Seven Cs of ASD. Like Predictive Parenting, content is adapted based on child verbal ability.

**Table 2:** Content covered in The Seven Cs of ASD

Group session	Content
1	Introduction and understanding ASD
2	**C**auses of ASD
3	**C**oncepts in ASD
4	**C**aring for yourself and your family: Part 1
5	**C**aring for yourself and your family: Part 2
6	**C**omorbidities in ASD: Part 1
7	**C**omorbidities in ASD: Part 2
8	**C**linical treatments for ASD
9	**C**ommunication and advocating for your child
10	**C**lassroom considerations
11	**C**aring for yourself and your family: Part 3
12	Recap and review

ASD, autism spectrum disorder.

### Intervention adherence

Detailed intervention manuals have been developed, and frequent clinical supervision is provided to reduce variability due to therapist effects. Checklists have been developed to measure intervention fidelity, which assess session content and group process. These are completed by the group facilitators after each intervention session.

### Sample size justification

As this is a pilot RCT, a formal sample size calculation was not undertaken. We are recruiting 60 families into the pilot RCT. We expect that retention will be approximately 90%, as reported by other trials of psychological intervention conducted with parents of young autistic children. We expect a more modest ES than the 1.3 reported by Sofronoff *et al* 19 as this was for a parent-reported measure and therefore unblinded. For the comparison of Predictive Parenting and the attention control condition, power was calculated by a non-central χ^2^ method using a linear mixed model with baseline (baseline-outcome correlation assumed 0.7) as covariate for two-tailed p=0.05, and intraclass correlation for within intervention group of 0.02 and 10% drop-out. For an ES of 0.5, our study has an expected 95% CI of 0.08, 0.92 and power of 64%, while for an ES of 0.6 the expected 95% CI is 0.18, 1.02 and 79% power.

### Outcomes

Table 3 displays measures that are being used in the trial and when they are administered.

**Table 3:** Administration of measures

Measure	Baseline	During treatment	Post intervention	Completed by
Primary outcome				
OSCA–AB child behaviours that challenge	✓		✓	Blinded researcher
**Secondary outcomes**				
OSCA–AB child compliance	✓		✓	Blinded researcher
OSCA–AB child-centered parenting behaviour	✓		✓	Blinded researcher
OSCA–AB child-directive parenting behaviour	✓		✓	Blinded researcher
ABC irritability and hyperactivity	✓		✓	Parent/teacher
ACB	✓		✓	Parent/teacher
HSQ-ASD	✓		✓	Parent
PASR	✓		✓	Parent
Improvement in parent-nominated target problems	✓		✓	Parent/blinded researcher
CGI-I	✓		✓	Parent/blinded researcher
APSI	✓		✓	Parent
CAPES-DD parent efficacy	✓		✓	Parent
SWEMWBS	✓		✓	Parent
PS	✓		✓	Parent
Adverse events			✓	Parent/blinded researcher
**Sample characterisation**				
Demographics	✓			Parent
SCQ-Lifetime	✓			Parent
ADOS–2	✓			Blinded researcher
ABAS–3	✓			Parent
**Intervention-related measures**				
Intervention attendance		✓		Clinician
Intervention satisfaction			✓	Parent
Intervention fidelity		✓		Clinician
**Health economics measures**				
ONS personal well-being	✓		✓	Parent
EQ-5D quality of life	✓		✓	Parent
CSRI	✓		✓	Parent/blinded researcher
Facilitator time use		✓		Clinician

ABAS–3, *Adaptive Behaviour Assessment System*, third edition; ABC, aberrant behaviour checklist; ACB, assessment of concerning behaviour; ADOS–2, *Autism Diagnostic Observation Schedule*, second edition; APSI, Autism Parenting Stress Index; CAPES-DD, Child Adjustment and Parent Efficacy Scale-Developmental Disability; CGI-I, clinical global impression-improvement; CSRI, Client Service Receipt Inventory; HSQ-ASD, Home Situations Questionnaire-Autism Spectrum Disorders; ONS, Office of National Statistics; OSCA–AB, Observation Schedule for Children with Autism-Anxiety and Behaviour; PASR, Preschool Anxiety Scale Revised; PS, Parenting Scale; SCQ, Social Communication Questionnaire; SWEMWBS, Short Warwick-Edinburgh Mental Well-being Scale.

#### Primary outcome

The primary outcome measure is child behaviours that challenge displayed during an observation of researcher–child and parent–child interactions. We have developed the Observation Schedule for Children with Autism–Anxiety and Behaviour (OSCA–AB) for the trial drawing on existing well-validated observational measures of parent– child interaction.^20–23^ Two researcher-led and six parent-led tasks are completed during the 18–22 min observation. Tasks aim to simulate everyday challenges that autistic children may face and find difficult. The frequency of a range of child behaviours that challenge (destructive behaviour, aggression towards themselves and others, frustrated vocalisations, non-compliance, avoidance and reassurance seeking) observed during the OSCA–AB are coded. As the length of the observation varies, the rate of child behaviours that challenge per minute is calculated. Further information about the measure will be published in a separate manuscript.

#### Secondary outcomes

##### Observed child compliance

The frequency of observed child compliance during the OSCA–AB is coded and the rate of child compliance per minute is calculated.

##### Observed parent behaviour

Frequencies of a range of observed parent behaviour (eg, positive and negative comments, commands, giving the child opportunity to comply, praise, physical handling and supportive physical guidance) during the OSCA–AB are coded and differences between groups will be examined. Child-centered parenting behaviours (positive comments, clear commands, praise and supportive physical guidance) and child-directive parenting behaviours (negative comments, unclear commands, no opportunity to comply and physical handling) are summed to produce total child-centered parenting and child-directive parenting scores. Due to variation in the length of the observation, rates of child-centered and child-directive parenting behaviours per minute are calculated. The proportion of child-centered parenting behaviour/child-centered and child-directive parenting behaviours is also calculated.

##### Parent-reported child emotional and behavioural difficulties

Parent-rated child emotional and behavioural difficulties is measured using The Aberrant Behaviour Checklist (ABC)^24^ Irritability and Hyperactivity subscales. The Assessment of Concerning Behaviours (ACB) scale,^25^ a measure of child mental health and concerning behaviours developed specifically for use with autistic individuals, is also completed. Forty-four items are rated on a 5-point sliding scale anchored by opposing responses (‘not at all’ and ‘very much’). The Home Situations Questionnaire-Autism Spectrum Disorders,^26^ an autism-specific measure of child non-compliance in everyday situations is also administered. Parent-reported child anxiety is measured using the Preschool Anxiety Scale Revised,^27^ which taps into specific fears, and generalised, social and separation anxiety.

A narrative describing one or two of the most pressing problems for parents related to child emotions and behaviours (parent-nominated target problems) is elicited at baseline. Information on the presentation, frequency, duration, intensity and interference with daily function, family life and other consequences is sought.^28^ The narratives are reviewed at post intervention and change from baseline is scored on a 9-point scale. The Clinical Global Impression-Improvement^29^ is used to rate overall improvement in child emotional and behavioural difficulties based on the parent-nominated target problems and parental perceptions of improvement.

##### Teacher-reported child emotional and behavioural difficulties

The ABC^24^ Irritability and Hyperactivity subscales are completed by the child’s teacher or someone involved in their education (eg, key worker and special educational needs co-ordinator). The teacher version of the ACB^25^ is also completed.

#### Parent-reported parenting outcomes

Parent-rated parenting stress associated with core and comorbid symptoms is measured using the Autism Parenting Stress Index^30^ and parenting self-efficacy is measured using the Child Adjustment and Parent Efficacy Scale-Developmental Disability Parent Efficacy subscale,^31^ a 16-item scale assessing confidence in managing specific child behaviours. The Short Warwick-Edinburgh Mental Wellbeing Scale^32^ assesses parent reports of their own well-being. The short version of the Parenting Scale^33^ is used to measure self-reported lax and overreactive parenting practices.

#### Sample characterisation measures

Demographic information about the family is obtained at baseline. Autism severity is measured at baseline only using the parent-reported Social Communication Questionnaire-Lifetime version,^34^ along with the *Autism Diagnostic Observation Schedule,* second edition (ADOS–2).^35^ The ADOS–2 is the gold standard observation for assessing autism symptoms and is administered by trained researchers. The *Adaptive Behaviour Assessment System*, third edition^36^ is completed by parents at baseline and measures three broad domains of adaptive skills and functioning (conceptual, social and practical), resulting in a General Adaptive Composite score.

#### Intervention-related measures

Attendance at intervention sessions and retention in the intervention are recorded. Satisfaction with the content and delivery of both interventions is measured using questionnaires developed for study.

#### Health economic measures

Parental well-being and daily emotions are measured using the Office of National Statistics (ONS) Personal Wellbeing questions,^37^ which ask about life satisfaction, worth, happiness and anxiety. The 5-level EQ-5D^38^ is used to measure parent reports of their own health-related quality of life, and index-based values are available to enable quality-adjusted life years (QALYs) calculations to be used in the cost-effectiveness analysis.

An adapted version of the Client Service Receipt Inventory (CSRI)^39^ measures service use and cost-related impacts at baseline and post intervention, to inform the cost-effectiveness analysis. Parents are asked to retrospectively identify all public, private and voluntary sector services used by the child, as well as services used by other family members that are linked to the child’s autism or emotional and behavioural difficulties. The CSRI also includes information on unpaid support and employment impacts on other family members. The facilitators delivering the interventions track their time spent on intervention-related activities and travel costs to be used in costing the interventions.

### Procedure

Children between the ages of 4 and 8 years with a diagnosis of ASD are recruited to the study from participating services following referral via local autism diagnostic teams, education professionals, support groups and consented databases. Potential participants can also self-refer. As the intervention content is adapted based on child verbal ability, the groups are run separately with parents of minimally verbal and verbal children within each of our localities. Therefore, the blocks of 10–18 families recruited for allocation to condition will be stratified by verbal ability level (minimally verbal: defined by ADOS–2 Module 1 vs verbal children: defined as ADOS–2 Module 2 or above) and by locality (Croydon, Bromley) as part of the recruitment procedure.

After initial contact and prescreening for eligibility, research staff obtain informed consent and conduct baseline assessments to confirm eligibility. All families are assigned a unique participant ID. Questionnaire measures are completed online or in hard copy depending on the parent’s preference. Other measures are completed during a visit to the research setting, over the phone or at the child’s school. Baseline assessments with families are conducted up to 2 months prior to randomisation. With parental consent, teachers are asked to complete questionnaires about the child’s emotional and behavioural difficulties at school. Post intervention assessments are conducted after the completion of the intervention. Outcome measures are sought for all families regardless of their participation in the treatment provided.

There are separate research and clinical teams who are based in different buildings and have separate supervision structures. The assessments and interventions are conducted in a way to avoid inadvertent divulging of information that could reveal allocation status. The location and materials used during the research assessments are different in type and location to those used for the intervention sessions, avoiding any familiarity effect for parents. Researchers involved in conducting the assessments and rating outcome measures are blinded to intervention content and participant condition. Group facilitators are blinded to primary outcome measurement.

### Data management, confidentiality and access

All data in the trial are anonymised. All paper records are filed anonymously by the participant’s unique study number in secure locked cabinets in the Department of Child and Adolescent Psychiatry, IoPPN, King’s College London. Consent forms are stored separately. Personal details (eg, name, address and telephone numbers) are stored in a separate encrypted database and linked by initial, date of birth and unique participant ID number. Some records from the feasibility phase are stored securely at York University.

Data from paper case report forms are entered on SPSS databases and along with other electronic data, stored on a King’s server folder that is accessible only to the research team. Double data entry will be completed on at least 10% of all entered data, and quality checks will be conducted. The principal investigator, trial statisticians and other members of the study team have access to final datasets and will undertake analysis as appropriate and necessary. Any arrangements for other researchers to have access to the data will be negotiated separately and the Central Office of Research Ethics Committee will be informed.

### Statistical analyses

A statistical analysis plan has been written by the trial statisticians (AP and DS) and will be approved by the chief investigator and the Data Monitoring Committee (DMC) prior to any analysis being undertaken. The analyses will be carried out using Stata.

In accordance with Consolidated Standards of Reporting Trials guidelines, we will report the flow of participants through the trial. Descriptive statistics of recruitment, drop-out and completeness of assessments and interventions will be provided. Satisfaction and fidelity of the intervention will also be reported descriptively. Baseline characteristics will be presented by group.

The main analysis will be via intention-to-treat, including all participants who were randomised. It will use statistical techniques for handling missing outcome data under a missing at random assumption, and multiple imputation for missing measures will be considered. We will test for a between-group change in the primary outcome at post intervention, using analysis of covariance (ANCOVA) regression predicting outcome where post intervention is also covaried for baseline. Dummy variables will be used to account for randomisation stratification and the clustering effects of groups. The distribution of the primary outcome at baseline will be examined for evidence of floor effects. Where floor effects are present, a generalised mixed model/structural equation modelling setup, in which both baseline and post intervention are modelled as potentially censored response variables, will be used with a covariance between equations that yield the ANCOVA estimate of treatment effect in the absence of censoring. Secondary outcome measures will be analysed in the same way. Analysis of all post intervention treatment effects will be undertaken after all post intervention outcome measures are completed. Trial statisticians will remain blinded until after the primary and secondary outcomes are analysed.

### Economic evaluation

The cost for each participant in the pilot will be derived by the product of the quantity of each service and support used and the unit cost of each of them. Unit costs will be based on the economic notion of opportunity costs—which considers the value of the resource in its next best alternative use. Where this is not practicable, unit costs will be approximated by nationally representative health and personal social services tariffs. Where unit costs are not readily available from such sources, we will derive costs using approaches outlined in an annual compendium of *Unit Cost of Health and Social Care*. We will use the most recent publication of the *Unit Cost of Health and Social Care* produced by the Personal Social Services Research Unit at the time of analysis. All other reported costs will be consistent with the price level used in that edition.^40^

When applying unit costs to unpaid care, we will use other approaches such as replacement costs. Under this approach, unpaid care by family and other carers will be costed using the average hourly rate for a local authority home care worker as the assumed cost for each hour of unpaid informal care.

Consistent with the outcome analyses, the economic evaluation will also conduct an intention-to-treat analysis, including all participants who were randomised. We will compute and compare comprehensively measured costs (for each of the two perspectives adopted: health and social care, public sector or societal) for the two interventions. Under each perspective, the cost-effectiveness analyses will bring together costs and the primary outcome and will compute indicative incremental cost-effectiveness ratios and net benefits; the societal perspective will be adopted in the main analyses. In a secondary economic evaluation, QALY gains computed from parental EQ-5D- scores will be compared with costs from each perspective; again, the societal perspective will be adopted to facilitate comparisons with the main analyses. Other exploratory cost-effectiveness analyses will examine other outcomes and perspectives.

In each case, an incremental cost-effectiveness ratio will be computed as the mean cost difference between Predictive Parenting and the attention control condition divided by the mean difference in change in measures of outcome respectively. If one treatment is indicating it is likely to be both more effective and costlier than the other, we would consider if it is worth incurring the higher costs in order to achieve the improved outcomes. The approach we will employ to reveal the nature of trade-offs such as these—and to represent the inherent uncertainty in any evaluation—will be to plot cost-effectiveness acceptability curves generated from bootstrap analyses. Sensitivity analyses will explore the impact of key assumptions such as the costing of unpaid care time and lost productivity, and the choice of outcome.

## Ethics and dissemination

The SPIRIT reporting guidelines are followed for this protocol.^41^

For the pilot RCT, we formed a Trial Steering Committee (TSC) which includes an independent chair, independent members and parent representatives (see below for membership). The TSC met prior to the commencement of the pilot RCT to agree the study protocol and will meet at least annually thereafter. The TSC were consulted on the study protocol, techniques for ascertainment and the focus of measurement including the primary outcome. They were also consulted on whether a DMC is required and decided that a sub-committee of the TSC (consisting of the chair and statistician) could act as the DMC.

Adverse events are measured at post intervention and include events related to child, parent and family well-being that may not be captured by outcome measures (eg, increased family discord, school refusal, significant change in a sibling’s well-being or behaviour) as well as predefined standard medical events. Such events that arise during treatment are documented when a situation becomes known to group facilitators. The TSC and DMC have independent oversight of the study and are informed of all adverse events.

This trial will contribute to the literature on parenting interventions for reducing emotional and behavioural difficulties displayed by young autistic children. As the study is a pilot RCT, conclusions about the efficacy of the intervention are not possible. However, the study design enables us to consider the feasibility of conducting a large-scale RCT to test the efficacy of Predictive Parenting. The findings from the pilot RCT will be disseminated through publication in peer-reviewed journals of general and special interest and presentations at national and international conferences. There will also be a general dissemination programme for families including participants co-ordinated through our collaborators in the National Autistic Society.

## Trial status

Protocol V.1.4, dated 04/02/2019, see our ISRCTN record for log of protocol amendments. Recruitment was completed on 16/10/2018. Post-intervention assessments are due for completion by 30/04/2019.

## Trial sponsor

King’s College London and South London and Maudsley NHS Foundation Trust. Email: slam-ioppn.research@kcl.ac.uk.

## Trial steering committee

Professor Alan Stein, University of Oxford (chair); Dr Matt Sydes, MRC Clinical Trials Unit, University College London (statistician, member); Dr Jacqueline Rodgers, University of Newcastle (member); Bridget Gilchrist (parent representative); Lindsay Stairs (parent representative).

## Data monitoring committee

As the trial is a pilot RCT, the TSC agreed that a subgroup consisting of Professor Alan Stein and Dr Matt Sydes would act as the DMC for ASTAR.

## Supplementary Material

Reviewer comments

Author's manuscript
