# Supplementary material for: A novel group parenting intervention to reduce emotional and behavioural difficulties in young autistic children: protocol for the Autism Spectrum Treatment and Resilience pilot randomised controlled trial
Source: BMJ Open. 2019 Jun 27;9(6):e029959. doi: 10.1136/bmjopen-2019-029959 (PMC6597639; doi:10.1136/bmjopen-2019-029959)
Supplement: Author's manuscript [file bmjopen-2019-029959.draft_revisions.pdf]

BMJ Open is committed to open peer review. As part of this commitment we make the peer review history of every article we publish publicly available.

When an article is published we post the peer reviewers' comments and the authors' responses online. We also post the versions of the paper that were used during peer review. These are the versions that the peer review comments apply to.

The versions of the paper that follow are the versions that were submitted during the peer review process. They are not the versions of record or the final published versions. They should not be cited or distributed as the published version of this manuscript.

BMJ Open is an open access journal and the full, final, typeset and author-corrected version of record of the manuscript is available on our site with no access controls, subscription charges or pay-per-view fees (http://bmjopen.bmj.com).

If you have any questions on BMJ Open's open peer review process please email info.bmjopen@bmj.com

BMJ Open

A novel group parenting intervention to reduce emotional and behavioural difficulties in young autistic children: Protocol for the Autism Spectrum Treatment and Resilience (ASTAR) pilot randomised controlled trial.

Journal:	*BMJ Open*
Manuscript ID	bmjopen-2019-029959
Article Type:	Protocol
Date Submitted by the Author:	20-Feb-2019

Complete List of Authors:	Palmer, Melanie; Institute of Psychiatry, Psychology & Neuroscience, King's College London, Department of Child and Adolescent Psychiatry<br>Tarver, Joanne; Aston University School of Life and Health Sciences, Department of Psychology; University of Birmingham, 3Cerebra Centre for Neurodevelopmental Disorders, School of Psychology<br>Paris Perez, Juan; Institute of Psychiatry, Psychology & Neuroscience, King's College London, Department of Child and Adolescent Psychiatry<br>Cawthorne, Thomas; Institute of Psychiatry, Psychology & Neuroscience, King's College London, Department of Child and Adolescent Psychiatry<br>Romeo, Renee; Institute of Psychiatry, Psychology & Neuroscience, King's College London, Department of Health Service and Population Research<br>Stringer, Dominic; Institute of Psychiatry, Psychology & Neuroscience, King's College London, Department of Biostatistics and Health Informatics<br>Hallett, Victoria; South London and Maudsley NHS Foundation Trust<br>Mueller, Joanne; South London and Maudsley NHS Foundation Trust<br>Breese, Lauren; South London and Maudsley NHS Foundation Trust<br>Hollett, Megan; South London and Maudsley NHS Foundation Trust<br>Beresford, Bryony; University of York, Social Policy Research Unit<br>Knapp, Martin; London School of Economics, Personal Social Services Research Unit<br>Slonims , Vicky; Guy's and St Thomas' NHS Foundation Trust, Evelina Children's Hospital<br>Pickles, Andrew; Institute of Psychiatry, Psychology & Neuroscience, King's College London, Department of Biostatistics and Health Informatics<br>Simonoff, Emily; Institute of Psychiatry, Psychology & Neuroscience, King's College London, Department of Child and Adolescent Psychiatry; South London and Maudsley NHS Foundation Trust<br>Scott, Stephen; Institute of Psychiatry, Psychology & Neuroscience, King's College London, Department of Child and Adolescent Psychiatry; South London and Maudsley NHS Foundation Trust<br>Charman, Tony; King's College London, Institute of Psychiatry, Department of Psychology; South London and Maudsley NHS Foundation Trust

Keywords:	Autism, Emotional and Behavioural Difficulties, Parenting Intervention,

	Feasibility, Pilot RCT

SCHOLARONE™
Manuscripts

TITLE: A novel group parenting intervention to reduce emotional and behavioural difficulties in young autistic children: Protocol for the Autism Spectrum Treatment and Resilience (ASTAR) pilot randomised controlled trial.

AUTHORS:

Melanie Palmer[1], Joanne Tarver[2,3], Juan Paris Perez[1], Thomas Cawthorne[1], Renee Romeo[1], Dominic Stringer[1], Victoria Hallett[4], Joanne Mueller[4], Lauren Breese[4], Megan Hollett[4], Bryony Beresford[5], Martin Knapp[6], Vicky Slonims[7], Andrew Pickles[1], Emily Simonoff[1,4], Stephen Scott[1,4] and Tony Charman[1,4].

AUTHOR AFFILIATIONS:

[1]King's College London, Institute of Psychiatry, Psychology & Neuroscience, London, UK.

[2]Department of Psychology, School of Life and Health Sciences, Aston University, Birmingham, UK

[3]Cerebra Centre for Neurodevelopmental Disorders, School of Psychology, University of Birmingham, Birmingham, UK

[4]South London and Maudsley NHS Foundation Trust, London, UK

[5]Social Policy Research Unit, University of York, York, UK

[6]Department of Health Policy, London School of Economics and Political Science, London, UK

[7]Newcomen Neurodevelopmental Centre, Children's Neurosciences, Evelina Children's Hospital, Guy's and St Thomas NHS Foundation Trust, London, UK.

For peer review only - http://bmjopen.bmj.com/site/about/guidelines.xhtml

CORRESPONDING AUTHOR:

The corresponding author, Melanie Palmer, can be contacted via email at melanie.palmer@kcl.ac.uk, or by telephone on +44 (0) 207 848 5260.

ORCID NUMBERS:

Melanie Palmer	0000-0001-5579-2170
Joanne Tarver	0000-0003-0555-6043
Juan Paris Perez	0000-0003-3171-0315
Thomas Cawthorne	0000-0003-4537-0016
Renee Romeo	0000-0003-3871-9697
Dominic Stringer	0000-0001-5624-1733
Victoria Hallett	0000-0002-7432-9824
Joanne Mueller	0000-0003-2737-1883
Lauren Breese	0000-0002-1246-7703
Megan Hollett	0000-0003-3123-1867
Bryony Beresford	0000-0003-0716-2902
Martin Knapp	0000-0003-1427-0215
Vicky Slonims	0000-0003-3339-2365
Andrew Pickles	0000-0003-1283-0346
Emily Simonoff	0000-0002-5450-0823
Stephen Scott	0000-0003-4680-6213
Tony Charman	0000-0003-1993-6549

For peer review only

ABSTRACT

Introduction: The majority of young autistic children display impairing emotional and behavioural difficulties that contribute to family stress. There is some evidence that behavioural parenting interventions are effective for reducing behavioural difficulties in autistic children, with less evidence assessing change in emotional difficulties. Previous trials have tended to use unblinded parent-report measures as primary outcomes and many do not employ an active control, limiting the conclusions that can be drawn.

Methods and analysis: The Autism Spectrum Treatment and Resilience (ASTAR) study is a pilot randomised controlled trial (RCT) testing the specific effect of a 12-week group parenting intervention (Predictive Parenting) on primary and secondary outcomes, in comparison to an attention control condition consisting of psychoeducation parent groups. Following a feasibility study to test research procedures and the interventions, the pilot RCT participants include 60 parents of 4-8 year old autistic children who are randomised to Predictive Parenting versus the attention control. Measures are administered at baseline and post-intervention to assess group differences in the child and parent outcomes, costs and service use, and adverse events. The primary outcome is an objective measure of child behaviour that challenges during interactions with their parent and a researcher. The trial aims to provide data on recruitment, retention, completion of measures and acceptability of the intervention and research protocol, in addition to providing a preliminary indication of potential efficacy and establishing an effect size that could be used to power a larger-scale efficacy trial. We will also provide preliminary estimates of the cost-effectiveness of the interventions.

Ethics and dissemination: Ethical approval was granted from NHS Camden and Kings Cross Research Ethics Committee (ref: 16/LO/1769) along with NHS R&D approval from South London and Maudsley, Guy's and St Thomas', and Croydon Health Services NHS

Trusts. The findings will be disseminated through publication in peer-reviewed journals and presentations at conferences.

Trial registration number: ISRCTN91411078.

Strengths and limitations of the study:

- The trial uses an objective measure as the primary outcome overcoming biases associated with participants being unblinded to treatment status.
- The target intervention, developed by clinicians with expertise in autism, is compared to an attention control condition to further guard against placebo effects.
- A feasibility study with nested qualitative evaluation enabled refinement of the intervention and research procedures prior to commencing the pilot RCT.
- Parents and autistic adults, referred to as patient and public involvement (PPI) panels, were involved in the development of the interventions and research procedures.
- As the study is a pilot RCT, conclusions about the efficacy of the intervention are not possible.

Keywords: Autism; Emotional and Behavioural Difficulties; Parenting Intervention; Feasibility; Pilot RCT.

For peer review only

INTRODUCTION

Background

Autism is characterised by difficulties in reciprocal social communication and the presence of restricted interests, repetitive behaviours and sensory anomalies.(1) At least 1% of children are autistic(2-4) and the condition is around three to four times more prevalent in males than females.(5) There are high rates of intellectual disability in autistic children with approximately 55% having an IQ below 70.(6) It has been demonstrated that additional psychiatric disorders frequently co-occur with autism at rates much higher than in the general population; up to 80-90% of young autistic children have additional emotional or behavioural difficulties meeting formal diagnostic criteria, with many having two or more additional disorders.(7-9) Anxiety disorders, attention deficit/hyperactivity disorder, and opposition defiant disorder are most common, and these difficulties tend to persist over time.(10)

Parents often report that it is these co-occurring difficulties, which are associated with poorer parental wellbeing and parental stress,(11) that they would like support with. Universal interventions are warranted given the high prevalence of co-occurring emotional and behavioural difficulties in autistic children. However, current service provision in the United Kingdom usually includes the offer of psychoeducation groups that focus on teaching parents about autism and developing strategies to support social and communication functioning, rather than the commonly co-occurring emotional and behavioural difficulties.

Behavioural parenting interventions are recommended by the National Institute of Health and Care Excellence(12) for the treatment of behavioural difficulties displayed by young children without autism. There are a number of effective parenting interventions that aim to reduce such difficulties in young autistic children. A recent meta-analysis of eight randomised controlled trials (RCTs) of behavioural parenting interventions aiming to reduce disruptive behaviour displayed by young autistic children(13) found a moderate effect on

For peer review only - http://bmjopen.bmj.com/site/about/guidelines.xhtml

disruptive behaviour when compared to controls (Standardised Mean Difference=-0.59, 95% confidence interval [CI] -0.88, -0.30). However, there was significant heterogeneity in the effect of parenting interventions on disruptive behaviour which may be due to sample size, mode of delivery and the focus and duration of treatment. Only one RCT included in the review involved anxiety management techniques even though anxiety disorders are the most common co-occurring psychiatric diagnoses in autism and "behaviour that challenges" is often described as an observable manifestation of anxiety.(14,15) A recent meta-analysis of 14 RCTs of cognitive behavioural therapy (CBT) interventions for anxiety in young autistic children, most of which included parental components, demonstrated that reductions in anxiety could be achieved.(16)

In addition, only one parenting intervention reviewed by Postorino et al.(13) included group-based sessions for parents, even though groups are more scalable and have the added benefit of providing a support network for parents. More than half of the included RCTs compared parenting interventions to a waitlist control or care as usual,(13) limiting conclusions that can be drawn about the effects as participants would not be blind to treatment allocation. Being unblinded to treatment allocation is particularly problematic when self-report measures are used as primary outcomes,(17) and there is a need for objective blinded measures of behaviour to be used as outcome measures in trials aiming to reduce emotional and behavioural difficulties displayed by young autistic children.

Aims and objectives

The Autism Spectrum Treatment and Resilience (ASTAR) trial is part of a research programme that aims to improve mental health outcomes among autistic individuals (Improving Autism Mental Health: https://iamhealthkcl.net/). ASTAR tests the specific effect of the Predictive Parenting intervention on child emotional and behavioural difficulties, in comparison to an attention control condition (psychoeducation parent groups). The aims of

For peer review only - http://bmjopen.bmj.com/site/about/guidelines.xhtml

the ASTAR trial are to: (1) examine the feasibility of the intervention in terms of recruitment, retention, completion of research measures and acceptability to parents; (2) provide a preliminary indication of potential efficacy on the primary and secondary outcomes and establish an effect size that could be used to power a future larger scale RCT; and (3) provide preliminary estimates of the cost-effectiveness of the intervention to inform a larger trial.

Consistent with Medical Research Council guidance on evaluating complex interventions,(18) we first conducted a preliminary feasibility phase testing the proposed research procedures and the Predictive Parenting (target intervention) and psychoeducation (control) group interventions with families with a 4-8 year old autistic child. A nested qualitative evaluation was conducted to explore the views of parents who declined to take part, those who completed/dropped-out of the interventions and the group facilitators. Findings from the feasibility phase were used to amend the research procedures and intervention manuals prior to the subsequent pilot RCT (see below for further information on learning from the feasibility phase).

The primary outcome of the pilot RCT is observed child behaviour that challenges, captured during a structured researcher- and parent-child interaction assessment (see description of measure below for further details). Secondary outcomes are child compliance and child-centred and child-directive parenting captured from the same observation and parent- and teacher-report of child emotional and behavioural difficulties. We are also measuring the effects of the interventions on parental stress and wellbeing, parenting practices and parenting self-efficacy.

METHODS AND ANALYSIS

Learning from the feasibility phase

The aim of the feasibility phase was to test the proposed recruitment processes and rates, the adequacy and acceptability of proposed measures and obtain the views of parents

For peer review only - http://bmjopen.bmj.com/site/about/guidelines.xhtml

For peer review only

and professionals on the research processes and interventions. Participants were 22 families (91% mothers and 9% fathers) with a 4-8 year old child with a clinical diagnosis of autism spectrum disorder. All but one of the children were male, and children were spilt across mainstream (*n*=10) and two special schools (*n*=12). Children in the special schools groups attended either a mixed autism-specific special school or a special school catering for children with severe learning difficulties co-occurring with autism. As intervention content is differentiated by child verbal ability, parents of minimally verbal children (*n*=12) attended groups separately from parents of verbal children (*n*=10).

We recruited 22 out of our target of 24 (92%) for the feasibility phase and we retained 20/22 (91%) families in the research protocol to post-intervention, indicating that the research processes were acceptable to families. All 22 parents gave consent for their child's teacher to complete measures. Baseline teacher questionnaires were obtained for 20/22 (91%) children and retention of teachers at post-intervention was high (18/22, 82%).

Parents who were interviewed reported that the research procedures were acceptable, although some felt the assessment process was lengthy. Prior to commencing the pilot RCT, two proposed outcome measures were removed to reduce burden on families (see our ISRCTN record for a log of outcome measures tested during the feasibility phase). For some parents, there appeared to have been a lack of clarity about the difference between the research and clinical teams and who they would have contact with at each stage of the study. This led to amendments in the information given to parents to help make this distinction clearer. Findings from the qualitative interviews indicated that most parents reported that they found the groups helpful and that they enjoyed meeting other parents in a similar situation. Feedback on the structure, timing, course materials and homework led to modifications to the Predictive Parenting intervention. For example, changes were made to make the groups more accessible and relevant to parents of children with lower levels of verbal ability. The study

For peer review only - http://bmjopen.bmj.com/site/about/guidelines.xhtml

design was also amended by increasing the number of families in each group (from six to eight) as it was a more efficient way to recruit and deliver the interventions. The increased group size was not thought to disrupt the intervention; indeed the slightly larger sizes may be helpful for group dynamics. Further details on the feasibility study can be provided upon contact with the research team.

Patient and Public Involvement (PPI)

Panels of parents of autistic children and autistic adults have been involved in all phases of the study and assisted with the development of the intervention curriculums and adaptions for parents of minimally verbal children, as well as advising on the research procedures. Guidance and advice about language to use when speaking with parents about the therapy goals and research processes (including on the written materials such as flyers and information sheets) was given.

Trial design

The study is a parallel group pilot RCT. Participating families are allocated to one of two treatment arms (Predictive Parenting or psychoeducational parent groups). Randomisation is conducted on blocks of 10-18 families on a ratio of 1:1, resulting in groups of 5-9 families in each treatment arm for any block. The randomisation algorithm is run by an independent statistician within the Biostatistics and Health Informatics Department, IoPPN, King's College London. Details of this are recorded in a separate randomisation specification document. Intervention allocation is emailed only to the group facilitators to ensure that the researchers are blind to condition.

Measures are collected at baseline, up to 2 months prior to the planned randomisation date, and approximately 18-24 weeks after randomisation once the 12-week intervention has finished. Group differences in outcomes will be examined.

Inclusion criteria

For peer review only - http://bmjopen.bmj.com/site/about/guidelines.xhtml

- Parent/carer of an autistic child, as confirmed by their clinician, aged between 4:0 years and 8:11 years
- Have sufficient spoken English to access the intervention
- Agree that their family doctor can be informed of their involvement in the trial.

Exclusion criteria

- Current participation in a behavioural parenting intervention delivered by another service
- Child has epileptic seizures more than weekly
- Parent or child has a severe hearing or visual impairment
- Active significant safeguarding concerns or a current severe parental psychiatric disorder
- Participation in the initial feasibility phase.

Interventions

Predictive Parenting (target intervention)

Predictive Parenting builds on behavioural parenting interventions, an evidence-based, well-accepted and cost-effective approach to targeting disruptive behaviour in children without autism.(12) It also incorporates well-established parent-mediated cognitive-behavioural therapy strategies for managing child anxiety.(16) It consists of 12 weekly 2-hour groups which extend parents' understanding of autism and associated difficulties and focus on supporting parents to understand and manage their child's emotions and behaviours (see Table 1 for content covered in Predictive Parenting). Techniques for helping parents prevent and reduce disruptive behaviour and anxiety are taught. It also includes content on promoting parental self-care and stress reduction. Content is adapted based on child verbal ability (minimally verbal vs. verbal). In addition to the 12 group sessions, two individual sessions are conducted- one between sessions 2 and 4 and the other between sessions 10 and

For peer review only

12. These individual sessions are up to 60 minutes long and aim to support individualisation and generalisation of the strategies for each family. The intervention is conducted in the community in local child and adolescent mental health services, libraries, or schools. Further information about Predictive Parenting will be published in a separate manuscript.

Table 1. Table displaying the content covered in Predictive Parenting

Group session	Content
1	Understanding ASD
2	Becoming a Behaviour Predictor
3	The Power of Planning
4	Predictably Positive Household
5	Clever Communication
6	Predictable Praise and Rewards
7	Managing Challenging Behaviour and Meltdowns
8	Predictable Parent Action Plans
9	Understanding Anxiety
10	Anxiety and Unpredictability Toolkit 1
11	Anxiety and Unpredictability Toolkit 2
12	Looking Forward and Looking After Yourself

Psychoeducational parent group (attention control condition)

The 'Seven Cs of ASD', the attention control condition, also consists of 12 weekly 2-hour groups that aim to provide psychoeducation and social support, whilst not providing specific guidance on managing behaviours or emotions. Table 2 below displays the content covered in each session of The Seven Cs of ASD. Like Predictive Parenting, content is adapted based on child verbal ability.

Table 2. Table displaying the content covered in The Seven Cs of ASD

Group session	Content
1	Introduction and understanding ASD
2	Causes of ASD
3	Concepts in ASD
4	Caring for yourself and your family: Part 1
5	Caring for yourself and your family: Part 2
6	Co-morbidities in ASD: Part 1
7	Co-morbidities in ASD: Part 2
8	Clinical treatments for ASD
9	Communication and advocating for your child

For peer review only - http://bmjopen.bmj.com/site/about/guidelines.xhtml

10	Classroom considerations
11	Caring for yourself and your family: Part 3
12	Recap and review

Intervention adherence

Detailed intervention manuals have been developed and frequent clinical supervision is provided to reduce variability due to therapist effects. Checklists have been developed to measure intervention fidelity, which assess session content and group process. These are completed by the group facilitators after each intervention session.

Sample size justification

As this is a pilot RCT, a formal sample size calculation was not undertaken. We are recruiting 60 families into the pilot RCT. We expect that retention will be approximately 90%, as reported by other trials of psychological intervention conducted with parents of young autistic children. We expect a more modest effect size than the 1.3 reported by Sofronoff et al.(19) as this was for a parent-reported measure and therefore unblinded. For the comparison of Predictive Parenting and the attention control condition, power was calculated by a non-central chi-square method using a linear mixed model with baseline (baseline-outcome correlation assumed 0.7) as covariate for two-tailed p=.05 and intraclass correlation for within intervention group of 0.02 and 10% drop-out. For an effect size (ES) of 0.5, our study has an expected 95% CI of 0.08, 0.92 and power of 64%, while for an ES of 0.6 the expected 95% CI is 0.18, 1.02 and 79% power.

Outcomes

Table 3 below displays measures that are being used in the trial and when they are administered.

Primary outcome

The primary outcome measure is child behaviour that challenges displayed during an observation of researcher-child and parent-child interactions. We have developed the

For peer review only - http://bmjopen.bmj.com/site/about/guidelines.xhtml

Observation Schedule for Children with Autism – Anxiety and Behaviour (OSCA–AB) for the trial drawing on existing well-validated observational measures of parent-child interaction.(20-23) Two researcher-led and six parent-led tasks are completed during the 20-25 minute observation. Tasks aim to simulate everyday challenges that autistic children may face and find difficult. The frequency of a range of child behaviour that challenges (destructive behaviour, aggression towards themselves and others, frustrated vocalisations, non-compliance, avoidance and reassurance seeking) observed during the OSCA–AB are coded. As the length of the observation varies, the rate of child behaviour that challenges per minute is calculated. Further information about the measure will be published in a separate manuscript.

Secondary outcomes

Observed child compliance

The frequency of observed child compliance during the OSCA–AB is coded and the rate of child compliance per minute is calculated.

Observed parent behaviour

Frequencies of a range of observed parent behaviour (e.g., positive and negative comments, commands, giving the child opportunity to comply, praise, physical handling and supportive physical guidance) during the OSCA-AB are coded and differences between groups will be examined. Child-centred parenting behaviours (positive comments, clear commands, praise and supportive physical guidance) and child-directive parenting behaviours (negative comments, unclear commands, no opportunity to comply and physical handling) are summed to produce total child-centred parenting behaviour and child-directive parenting behaviour scores. Due to variation in the length of the observation, rates of child-centred and child-directive parenting behaviours per minute are calculated. The proportion of child-

For peer review only - http://bmjopen.bmj.com/site/about/guidelines.xhtml

centred parenting behaviour / child-centred and child-directive parenting behaviours is also calculated.

Parent-reported child emotional and behavioural difficulties

Parent-rated child emotional and behavioural difficulties is measured using The Aberrant Behaviour Checklist (ABC)(24) Irritability and Hyperactivity subscales. The Assessment of Concerning Behaviours (ACB) scale,(25) a measure of child mental health and concerning behaviours developed specifically for use with autistic individuals, is also completed. Forty-four items are rated on a 5-point sliding scale anchored by opposing responses ('not at all' and 'very much'). The Home Situations Questionnaire-Autism Spectrum Disorders (HSQ-ASD),(26) an autism-specific measure of child non-compliance in everyday situations is also administered. Parent-reported child anxiety is measured using the Preschool Anxiety Scale Revised (PASR),(27) which taps into specific fears, and generalised, social and separation anxiety.

A narrative describing one or two of the most pressing problems for parents related to child emotions and behaviours (Parent-Nominated Target Problems) is elicited at baseline. Information on the presentation, frequency, duration, intensity and interference with daily function, family life and other consequences is sought.(28) The narratives are reviewed at post-intervention and change from baseline is scored on a 9-point scale. The Clinical Global Impression-Improvement (CGI-I)(29) is used to rate overall improvement in child emotional and behavioural difficulties based on the parent-nominated target problems and parental perceptions of improvement.

Teacher-reported child emotional and behavioural difficulties

The ABC(24) Irritability and Hyperactivity subscales is completed by the child's teacher or someone involved in their education (e.g., key worker, Special Educational Needs Co-ordinator). The teacher version of the ACB(25) is also completed.

For peer review only - http://bmjopen.bmj.com/site/about/guidelines.xhtml

Parent-reported parenting outcomes

Parent-rated parenting stress associated with core and co-morbid symptoms is measured using the Autism Parenting Stress Index (APSI)(30) and parenting self-efficacy is measured using the Child Adjustment and Parent Efficacy Scale-Developmental Disability (CAPES-DD) Parent Efficacy subscale,(31) a 16-item scale assessing confidence in managing specific child behaviours. The Short Warwick-Edinburgh Mental Wellbeing Scale (SWEMWBS)(32) assesses parent reports of their own wellbeing. The short version of the Parenting Scale (PS)(33) is used to measure self-reported lax and overreactive parenting practices.

Sample characterisation measures

Demographic information about the family is obtained at baseline. Autism severity is measured at baseline only using the parent-reported Social Communication Questionnaire-Lifetime version (SCQ-L)(34), along with the ADOS–2.(35) The ADOS–2 is the gold standard observation for assessing autism symptoms and is administered by trained researchers. The Adaptive Behaviour Assessment System – 3rd edition (ABAS–3)(36) is completed by parents at baseline and measures three broad domains of adaptive skills and functioning (conceptual, social and practical), resulting in a General Adaptive Composite score.

Intervention related measures

Attendance at intervention sessions and retention in the intervention is recorded. Satisfaction with the content and delivery of both interventions is measured using questionnaires developed for study.

Health economic measures

Parental wellbeing and daily emotions are measured using the Office of National Statistics (ONS) Personal Wellbeing questions(37) which ask about life satisfaction, worth,

For peer review only - http://bmjopen.bmj.com/site/about/guidelines.xhtml

happiness, and anxiety. The EQ-5D-5L(38) is used to measure parent reports of their own health-related quality of life, and index-based values are available to enable quality-adjusted life years (QALYs) calculations to be used in the cost-effectiveness analysis.

An adapted version of the Client Service Receipt Inventory (CSRI)(39) measures service use and cost-related impacts at baseline and post-intervention, to inform the cost-effectiveness analysis. Parents are asked to retrospectively identify all public, private and voluntary sector services used by the child, as well as services used by other family members that are linked to the child's autism or emotional and behavioural difficulties. The CSRI also includes information on unpaid support and employment impacts on other family members. The facilitators delivering the interventions track their time spent on intervention-related activities and travel costs to be used in costing the interventions.

Table 3. Table showing administration of measures.

Measure	Baseline	During treatment	Post-intervention	Completed by
Primary outcome				
OSCA–AB Child Behaviour That Challenges	✓		✓	Blinded researcher
Secondary outcomes				
OSCA–AB Child Compliance	✓		✓	Blinded researcher
OSCA–AB Child-Centred Parenting Behaviour	✓		✓	Blinded researcher
OSCA–AB Child-Directive Parenting Behaviour	✓		✓	Blinded researcher
ABC Irritability and Hyperactivity	✓		✓	Parent/teacher
ACB	✓		✓	Parent/teacher
HSQ-ASD	✓		✓	Parent
PASR	✓		✓	Parent
Improvement in Parent-Nominated Target Problems	✓		✓	Parent/blinded researcher
CGI-I	✓		✓	Parent/blinded researcher
APSI	✓		✓	Parent

For peer review only - http://bmjopen.bmj.com/site/about/guidelines.xhtml

CAPES-DD Parent Efficacy	✓		✓	Parent
SWEMWBS	✓		✓	Parent
PS	✓		✓	Parent
Adverse events			✓	Parent/blinded researcher
Sample characterisation				
Demographics	✓			Parent
SCQ-Lifetime	✓			Parent
ADOS-2	✓			Blinded researcher
ABAS-3	✓			Parent
Intervention related measures				
Intervention attendance		✓		Clinician
Intervention satisfaction			✓	Parent
Intervention fidelity		✓		Clinician
Health economics measures				
ONS Personal Wellbeing	✓		✓	Parent
EQ-5D-5L Quality of Life	✓		✓	Parent
CSRI	✓		✓	Parent/blinded researcher
Facilitator time use		✓		Clinician

Note.
ABAS–3=Adaptive Behaviour Assessment System – 3rd edition; ABC=Aberrant Behaviour Checklist; ACB=Assessment of Concerning Behaviour; ADOS–2=Autism Diagnostic Observation Schedule – 2nd edition; APSI=Autism Parenting Stress Index; CAPES-DD=Child Adjustment and Parent Efficacy Scale-Developmental Disability; CGI-I=Clinical Global Impression-Improvement; CSRI= Client Service Receipt Inventory; HSQ-ASD=Home Situations Questionnaire-Autism Spectrum Disorders; ONS=Office of National Statistics; OSCA–AB=Observation Schedule for Children with Autism – Anxiety and Behaviour; PASR= Preschool Anxiety Scale Revised; PS= Parenting Scale; SWEMWBS=Short Warwick-Edinburgh Mental Wellbeing Scale; SCQ=Social Communication Questionnaire.

For peer review only

Procedure

Children between the ages of 4 and 8 years with a diagnosis of autism spectrum disorder (ASD) are recruited to the study from participating services following referral via local autism diagnostic teams, education professionals, support groups and consented databases. Potential participants can also self-refer. As the intervention content is adapted based on child verbal ability, the groups are run separately with parents of minimally verbal

For peer review only - http://bmjopen.bmj.com/site/about/guidelines.xhtml

and verbal children within each of our localities. Therefore, the blocks of 10-18 families recruited for allocation to condition will be stratified by verbal ability level (minimally verbal [defined as Autism Diagnostic Observation Schedule – 2nd edition, ADOS–2(35) Module 1] vs. verbal children [defined as ADOS–2 Module 2 or above]) and by locality (Croydon, Bromley) as part of the recruitment procedure.

After initial contact and pre-screening for eligibility, research staff obtain informed consent and conduct baseline assessments to confirm eligibility. All families are assigned a unique participant ID. Questionnaire measures are completed online or in hard copy depending on the parent's preference. Other measures are completed during a visit to the research setting, over the phone or at the child's school. Baseline assessments with families are conducted up to 2 months prior to randomisation. With parental consent, teachers are asked to complete questionnaires about the child's emotional and behavioural difficulties at school. Post-intervention assessments are conducted after the completion of the intervention. Outcome measures are sought for all families regardless of their participation in the treatment provided.

There are separate research and clinical teams who are based in different buildings and have separate supervision structures. The assessments and interventions are conducted in a way to avoid inadvertent divulging of information that could reveal allocation status. The location and materials used during the research assessments are different in type and location to those used for the intervention sessions, avoiding any familiarity effect for parents. Researchers involved in conducting the assessments and rating outcome measures are blind to intervention content and participant condition. Group facilitators are blind to primary outcome measurement.

Data management, confidentiality and access

For peer review only - http://bmjopen.bmj.com/site/about/guidelines.xhtml

All data in the trial are anonymised. All paper records are filed anonymously by the participant's unique study number in secure locked cabinets in the Department of Child and Adolescent Psychiatry, IoPPN, King's College London. Consent forms are stored separately. Personal details (e.g., name, address, telephone numbers) are stored in a separate encrypted database and linked by initial, date of birth and unique participant ID number. Some records from the feasibility phase are stored securely at York University.

Data from paper case report forms are entered on SPSS databases and along with other electronic data, stored on a King's server folder that is only accessible to the research team. Double data entry will be completed on at least 10% of all entered data and quality checks will be conducted. The principal investigator, trial statisticians and other members of the study team have access to final datasets and will undertake analysis as appropriate and necessary. Any arrangements for other researchers to have access to the data will be negotiated separately and the Central Office of Research Ethics Committee will be informed.

Statistical analyses

A statistical analysis plan has been written by the trial statisticians (AP and DS) and approved by the chief investigator, the Trial Steering Committee (TSC) and the Data Monitoring Committee (DMC) prior to any analysis being undertaken. The analyses will be carried out using Stata.

In accordance with CONSORT guidelines, we will report the flow of participants through the trial. Descriptive statistics of recruitment, drop-out and completeness of assessments and interventions will be provided. Satisfaction and fidelity of the intervention will also be reported descriptively. Baseline characteristics will be presented by group.

The main analysis will be via intention-to-treat, including all participants who were randomised. It will use statistical techniques for handling missing outcome data under a missing at random assumption and multiple imputation for missing measures will be

For peer review only - http://bmjopen.bmj.com/site/about/guidelines.xhtml

considered. We will test for a between-group change in the primary outcome at post-intervention, using ANCOVA regression predicting outcome where post-intervention is also covaried for baseline. Dummy variables will be used to account for randomisation stratification and the clustering effects of groups. The distribution of the primary outcome at baseline will be examined for evidence of floor effects. Where floor effects are present, a generalised mixed model/structural equation modelling setup, in which both baseline and post-intervention are modelled as potentially censored response variables, will be used with a covariance between equations that yields the ANCOVA estimate of treatment effect in the absence of censoring. Secondary outcome measures will be analysed in the same way. Analysis of all post-intervention treatment effects will be undertaken after all post-intervention outcome measures are completed. Trial statisticians will remain blind until after the primary and secondary outcomes are analysed.

Economic evaluation

The cost for each participant in the pilot will be derived by the product of the quantity of each service and support used and the unit cost of each of them. Unit costs will be based on the economic notion of opportunity costs – which considers the value of the resource in its next best alternative use. Where this is not practicable, unit costs will be approximated by nationally representative health and personal social services tariffs. Where unit costs are not readily available from such sources, we will derive costs using approaches outlined in an annual compendium of Unit Cost of Health and Social Care. We will use the most recent publication of the *Unit Cost of Health and Social Care* produced by the Personal Social Services Research Unit at the time of analysis. All other reported costs will be consistent with the price level used in that edition.(40)

When applying unit costs to unpaid care, we will use other approaches such as replacement costs. Under this approach, unpaid care by family and other carers will be costed

For peer review only - http://bmjopen.bmj.com/site/about/guidelines.xhtml

using the average hourly rate for a local authority home care worker as the assumed cost for each hour of unpaid informal care.

Consistent with the outcome analyses, the economic evaluation will also conduct an intention-to-treat analysis, including all participants who were randomised. We will compute and compare comprehensively measured costs (for each of the two perspectives adopted: health and social care, public sector or societal) for the two interventions. Under each perspective, the cost-effectiveness analyses will bring together costs and the primary outcome and will compute indicative incremental cost-effectiveness ratios and net benefits; the societal perspective will be adopted in the main analyses. In a secondary economic evaluation, QALY gains computed from parental EQ-5D-5L scores will be compared with costs from each perspective; again, the societal perspective will be adopted to facilitate comparisons with the main analyses. Other exploratory cost-effectiveness analyses will examine other outcomes and perspectives.

In each case, an incremental cost-effectiveness ratio will be computed as the mean cost difference between Predictive Parenting and the attention control condition divided by the mean difference in change in measures of outcome respectively. If one treatment is indicating it is likely to be both more effective and costlier than the other, we would consider if there is some suggestion that it is worth incurring the higher costs in order to achieve the improved outcomes. The approach we will employ to reveal the nature of trade-offs such as these – and to represent the inherent uncertainty in any evaluation – will be to plot cost-effectiveness acceptability curves generated from bootstrap analyses. Sensitivity analyses will explore the impact of key assumptions such as the costing of unpaid care time and lost productivity, and the choice of outcome.

ETHICS AND DISSEMINATION

For peer review only - http://bmjopen.bmj.com/site/about/guidelines.xhtml

Ethical approval was granted from NHS Camden and Kings Cross Research Ethics Committee on 18/11/2016 (ref: 16/LO/1769). Written consent is obtained from all participating parents. Assent from children is obtained where appropriate. The SPIRIT reporting guidelines are followed for this protocol.(41)

For the pilot RCT, we formed a TSC which includes an independent chair, independent members and parent representatives (see below for membership). The TSC met prior to the commencement of the pilot RCT to agree the study protocol and will meet at least annually thereafter. The TSC were consulted on the study protocol, techniques for ascertainment and the focus of measurement including the primary outcome. They were also consulted on whether a DMC is required and decided that a sub-committee of the TSC (consisting of the chair and statistician) could act as the DMC.

Adverse events are measured at post-intervention and include events related to child, parent and family wellbeing that may not be captured by outcome measures (e.g., increased family discord, school refusal, significant change in a sibling's wellbeing or behaviour) as well as pre-defined standard medical events. Such events that arise during treatment are documented when a situation becomes known to group facilitators. The TSC and DMC have independent oversight of the study and are informed of all adverse events.

This trial will contribute to the literature on parenting interventions for reducing emotional and behavioural difficulties displayed by young autistic children. As the study is a pilot RCT, conclusions about the efficacy of the intervention are not possible. However, the study design enables us to consider the feasibility of conducting a large-scale RCT to test the efficacy of Predictive Parenting. The findings from the pilot RCT will be disseminated through publication in peer-reviewed journals of general and special interest and presentations at national and international conferences. There will also be a general

For peer review only - http://bmjopen.bmj.com/site/about/guidelines.xhtml

dissemination programme for families including participants co-ordinated through our collaborators in the National Autistic Society.

For peer review only

For peer review only - http://bmjopen.bmj.com/site/about/guidelines.xhtml

TRIAL STATUS

Protocol version 1.4, dated 04/02/2019, see our ISRCTN record for log of protocol amendments. Recruitment was completed on the 16/10/2018. Post-intervention assessments are due for completion by 30/04/2019.

TRIAL SPONSOR

King's College London and South London and Maudsley NHS Foundation Trust. Email: slam-ioppn.research@kcl.ac.uk.

TRIAL STEERING COMMITTEE

Professor Alan Stein, University of Oxford (Chair); Dr Matt Sydes, MRC Clinical Trials Unit, University College London (Member); Dr Jacqueline Rodgers, University of Newcastle (Member); Bridget Gilchrist (Parent Representative); Lindsay Stairs (Parent Representative).

DATA MONITORING COMMITTEE

As the trial is a pilot RCT, the TSC agreed that a subgroup consisting of Professor Alan Stein and Dr Matt Sydes would act as the DMC for ASTAR.

ACKNOWLEDGEMENTS

We are grateful to the families who were involved in the initial feasibility study and to those who are involved in the pilot trial. We would like to thank other members of the IAMHealth consortium for their comments and advice on the trial design. We would also like to thank local professionals who assisted with identifying potential participants, with particular thanks to Dr Shade Alu, Sarran Bond, Marion Drennan, Dr Mark O'Leary, Dr Fernando Salazar and Jackie Sutherland. We would also like to acknowledge Katherine Appleby, Elena Baker,

For peer review only

Emma Biggin, Sophie Carruthers, Margot Frayne, Lydia Johnson-Ferguson, Moriya Maccabee and Sophie Webb for their assistance with data collection.

AUTHORS' CONTRIBUTIONS

MP, JT, JPP, RR, DS, BB, MK, VS, AP, ES, SS and TC were involved in designing the study and drafting the protocol for the pilot RCT. TCa is involved in recruiting and collecting data for the pilot RCT. VH, JM, LB and MH are involved in developing and delivering the interventions. The manuscript was drafted by MP and all authors read, made revisions and approved the final version.

FUNDING STATEMENT

This trial summarises independent research funded by the National Institute for Health Research (NIHR) under its Programme Grants for Applied Research programme (RP-PG-1211-20016). The views expressed in this presentation are those of the authors and not necessarily those of the NHS, the NIHR or the Department of Health. Additional funding for intervention materials was received from the Maudsley Charity (1157). MK receives support from the NIHR School for Social Care Research. AP receives support from the NIHR through a Senior Investigator Award (NF-SI-0617-10120). ES additionally receives support from the National Institute for Health Research (NIHR) Biomedical Research Centre at South London and Maudsley Foundation Trust (IS-BRC-1215-20018), the NIHR through a Senior Investigator Award (NF-SI-0514-10073), the European Union Innovative Medicines Initiative (EU-IMI 115300), Autistica (7237), Medical Research Council (MR/R000832/1, MR/P019293/1), the Economic and Social Research Council (ESRC 003041/1), Guy's and St Thomas' Charitable Foundation (GSTT EF1150502) and the Maudsley Charity. TC receives

For peer review only - http://bmjopen.bmj.com/site/about/guidelines.xhtml

grant or research support from the NIHR, the Medical Research Council, the European Union (IMI, H2020), Autistica, MQ and The Waterloo Foundation.

COMPETING INTERESTS

AP declares that he receives royalties from WPS for the Social Communication Questionnaire.

PATIENT CONSENT

Obtained.

ETHICAL APPROVAL

Obtained from NHS Camden and Kings Cross Research Ethics Committee (ref: 16/LO/1769).

PROVENANCE AND PEER REVIEW

Not commissioned; externally peer reviewed for funding and subsequently ethical approval prior to submission.

For peer review only

REFERENCES

(1) American Psychiatric Association. Diagnostic and Statistical Manual of Mental Disorders: DSM-5 (5th ed.). Arlington, VA: American Psychiatric Association 2013.

(2) Baird G, Simonoff E, Pickles A, et al. Prevalence of disorders of the autism spectrum in a population cohort of children in South Thames: the Special Needs and Autism Project (SNAP). *Lancet* 2006;368:210–215.

(3) Baio J, Wiggins L, Christensen DL, et al. Prevalence of autism spectrum disorder among children aged 8 years - Autism and Developmental Disabilities Monitoring Network, 11 Sites, United States, 2014. *MMWR Surveill* 2018;67:1–23.

(4) Russell G, Rodgers LR, Ukoumunne OC, et al. Prevalence of parent-reported ASD and ADHD in the UK: Findings from the Millennium Cohort Study. *J Autism Dev Disord* 2014;44:31–40.

(5) Loomes R, Hull L, Mandy WPL. What is the male-to-female ratio in autism spectrum disorder? A systematic review and meta-analysis. *J Am Acad Child Adolesc Psychiatry* 2017 ;56:466–474.

(6) Charman T, Pickles A, Simonoff E, et al. IQ in children with autism spectrum disorders: data from the Special Needs and Autism Project (SNAP). *Psychol Med* 2011;41:619–627.

(7) Salazar F, Baird G, Chandler S, et al. Co-occurring psychiatric disorders in preschool and elementary school-aged children with autism spectrum disorder. *J Autism Dev Disord* 2015;40:1080–1093.

(8) Chandler S, Howlin P, Simonoff E, et al. Emotional and behavioural problems in young children with autism spectrum disorder. *Dev Med Child Neurol* 2016;58:202–208.

(9) Simonoff E, Pickles A, Charman T, et al. Psychiatric disorders in children with autism spectrum disorders: Prevalence, comorbidity, and associated factors in a population-derived sample. *J Am Acad Child Adolesc Psychiatry* 2008;47:921–929.

(10) Simonoff E, Jones C R, Baird G, et al. The persistence and stability of psychiatric problems in adolescents with autism spectrum disorders. *J Child Psychol Psychiatry* 2013;54:186–194.

(11) Yorke I, White P, Weston A, et al. The association between emotional and behavioral problems in children with autism spectrum disorder and psychological distress in their parents: A systematic review and meta-analysis. *J Autism Dev Disord* 2018;48:3393–3415.

(12) National Institute for Health and Clinical Excellence. Antisocial behaviour and conduct disorders in children and young people: Recognition and management (CG158). 2013. https://www.nice.org.uk/guidance/cg158

For peer review only

For peer review only - http://bmjopen.bmj.com/site/about/guidelines.xhtml

(13) Postorino V, Sharp WG, McCracken CE, et al. A systematic review and meta-analysis of parent training for disruptive behavior in children with autism spectrum disorder. *Clin Child Fam Psychol Rev* 2017;20:391–402.

(14) Ozsivadjian A, Knott FF, Magiati I. Parent and child perspectives on the nature of anxiety in children and young people with autism spectrum disorders: A focus group study. *Autism* 2012;16:107–121.

(15) Bearss K, Taylor CA, Aman MG, et al. Using qualitative methods to guide scale development for anxiety in youth with autism spectrum disorder. *Autism* 2015;20:663–672.

(16) Ung D, Selles R, Small BJ, et al. A systematic review and meta-analysis of cognitive-behavioral therapy for anxiety in youth with high-functioning autism spectrum disorders. *Child Psychiatry Hum Dev* 2015;46:533–547.

(17) Sonuga-Barke EJ, Brandeis D, Cortese S, et al. Nonpharmacological interventions for ADHD: Systematic review and meta-analyses of randomized controlled trials of dietary and psychological treatments. *Am J Psychiatry* 2013;170:275–289.

(18) Craig P, Dieppe P, Macintyre S, et al. Developing and evaluating complex interventions: The new Medical Research Council guidance. *BMJ* 2008;337:a1655.

(19) Sofronoff K, Attwood T, Hinton S. A randomised controlled trial of a CBT intervention for anxiety in children with asperger syndrome. *J Child Psychol Psychiatry* 2005;46:1152–1160.

(20) Wakschlag LS, Hill C, Carter AS, et al. Observational assessment of preschool disruptive behavior, part I: Reliability of the Disruptive Behavior Diagnostic Observation Schedule (DB-DOS). *J Am Acad Child Adolesc Psychiatry* 2008;47:622–631.

(21) Robinson EA, Eyberg SM. The dyadic parent-child interaction coding system: Standardization and validation. *J Consult Clin Psychol* 1981;49:245–250.

(22) Johnson CR, Butter EM, Handen BL et al. Standardised Observation Analogue Procedure (SOAP) for assessing parent and child behaviours in clinical trials. *J Intellect Dev Disabil* 2009;34:230–238.

(23) Mian ND, Carter AS, Pine DS, et al. Development of a novel observational measure for anxiety in young children: The Anxiety Dimensional Observation Scale. *J Child Psychol Psychiatry* 2015;56:1017–1025.

(24) Aman MG, Singh NN, Stewart AW, et al. The Aberrant Behavior Checklist: A behavior rating scale for the assessment of treatment effects. *Am J Ment Defic* 1985;89:485–491.

(25) Santosh P, Tarver J, Gibbons F, et al. Protocol for the development and validation of a questionnaire to assess concerning behaviours and mental health in individuals with autism spectrum disorders: The Assessment of Concerning Behaviour (ACB) scale. *BMJ Open* 2016;6:e010693.

For peer review only

For peer review only - http://bmjopen.bmj.com/site/about/guidelines.xhtml

(26) Chowdhury M, Aman MG, Lecavalier L, et al. Factor structure and psychometric properties of the revised Home Situations Questionnaire for autism spectrum disorder: The Home Situations Questionnaire-Autism Spectrum Disorder. *Autism* 2015;54:281–291.

(27) Edwards SL, Rapee RM, Kennedy SJ, et al. The assessment of anxiety symptoms in preschool-aged children: The revised Preschool Anxiety Scale. *J Clin Child Adolesc Psychol* 2010;39:400–409.

(28) Arnold LE, Vitiello B, McDougle C, et al. Parent-defined target symptoms respond to risperidone in RUPP autism study: Customer approach to clinical trials. *J Am Acad Child Adolesc Psychiatry* 2003;42:1443-1450.

(29) Guy W. ECDEU Assessment Manual for Psychopharmacology: Publication ADM 76-338. 1976.

(30) Silva LM, Schalock M. Autism Parenting Stress Index: Initial psychometric evidence. *J Autism Dev Disord* 2012;42:566–574.

(31) Emser TS, Mazzucchelli TG, Christiansen H, et al. Child Adjustment and Parent Efficacy Scale-Developmental Disability (CAPES-DD): First psychometric evaluation of a new child and parenting assessment tool for children with a developmental disability. *Res Dev Disabil* 2016;53-54:158–177.

(32) NHS Health Scotland, University of Warwick and University of Edinburgh. Short Warwick Edinburgh Mental Well-Being Scale (SWEMWBS). 2008. https://warwick.ac.uk/fac/sci/med/research/platform/wemwbs/development/swemwbs/

(33) Arnold DS, O'Leary SG, Wolff LS, et al. The Parenting Scale: A measure of dysfunctional parenting in discipline situations. *Psychol Assess* 1993;5:137–144.

(34) Rutter M, Bailey A, Lord C. Social Communication Questionnaire. Los Angeles, CA: Western Psychological Services 2003.

(35) Lord C, Rutter M, DiLavore PC, et al. Autism Diagnostic Observation Schedule (2nd ed.). Torrance, CA: Western Psychological Services 2012.

(36) Harrison P, Oakland T. Adaptive Behavior Assessment System (ABAS-3) (3rd ed.) Los Angeles, CA: Western Psychological Services 2015.

(37) Office of National Statistics. Measuring national well-being: Personal wellbeing in the UK, 2014 to 2015 (Statistical Bulletin). 2015. https://www.ons.gov.uk/peoplepopulationandcommunity/wellbeing/bulletins/measuringnationalwellbeing/2015-09-23

(38) EuroQol Group. EuroQol--A new facility for the measurement of health-related quality of life. *Health Policy* 1990;16:199–208.

(39) Beecham J, Knapp M. Costing psychiatric interventions. In: Thornicroft G, ed. Measuring Mental Health Needs (2nd ed). London: Royal College of Psychiatrists 2001:200–224.

For peer review only - http://bmjopen.bmj.com/site/about/guidelines.xhtml

(40) Curtis L, Burns A. Unit costs of health and social care 2017. Personal Social Service Research Unit, University of Kent, Canterbury 2017. https://www.pssru.ac.uk/project-pages/unit-costs/unit-costs-2017/

(41) Chan AW, Tetzlaff JM, Altman DG, et al. SPIRIT 2013 Statement: Defining standard protocol items for clinical trials. *Rev Panam Salud Publica* 2015;38:506–514.

For peer review only

For peer review only - http://bmjopen.bmj.com/site/about/guidelines.xhtml

Reporting checklist for protocol of a clinical trial.

Based on the SPIRIT guidelines.

Instructions to authors

Complete this checklist by entering the page numbers from your manuscript where readers will find each of the items listed below.

Your article may not currently address all the items on the checklist. Please modify your text to include the missing information. If you are certain that an item does not apply, please write "n/a" and provide a short explanation.

Upload your completed checklist as an extra file when you submit to a journal.

In your methods section, say that you used the SPIRIT reporting guidelines, and cite them as:

Chan A-W, Tetzlaff JM, Altman DG, Laupacis A, Gøtzsche PC, Krleža-Jerić K, Hróbjartsson A, Mann H, Dickersin K, Berlin J, Doré C, Parulekar W, Summerskill W, Groves T, Schulz K, Sox H, Rockhold FW, Rennie D, Moher D. SPIRIT 2013 Statement: Defining standard protocol items for clinical trials. Ann Intern Med. 2013;158(3):200-207

		Reporting Item	Page Number
Title	#1	Descriptive title identifying the study design, population, interventions, and, if applicable, trial acronym	1
Trial registration	#2a	Trial identifier and registry name. If not yet registered, name of intended registry	4

For peer review only

Trial registration: data set	#2b	All items from the World Health Organization Trial Registration Data Set	1-26
Protocol version	#3	Date and version identifier	24
Funding	#4	Sources and types of financial, material, and other support	25-26
Roles and responsibilities: contributorship	#5a	Names, affiliations, and roles of protocol contributors	25
Roles and responsibilities: sponsor contact information	#5b	Name and contact information for the trial sponsor	24
Roles and responsibilities: sponsor and funder	#5c	Role of study sponsor and funders, if any, in study design; collection, management, analysis, and interpretation of data; writing of the report; and the decision to submit the report for publication, including whether they will have ultimate authority over any of these activities	24-25
Roles and responsibilities: committees	#5d	Composition, roles, and responsibilities of the coordinating centre, steering committee, endpoint adjudication committee, data management team, and other individuals or groups overseeing the trial, if applicable (see Item 21a for data monitoring committee)	19, 22, 24

For peer review only

Background and rationale	#6a	Description of research question and justification for undertaking the trial, including summary of relevant studies (published and unpublished) examining benefits and harms for each intervention	5-7
Background and rationale: choice of comparators	#6b	Explanation for choice of comparators	5-6
Objectives	#7	Specific objectives or hypotheses	6-7
Trial design	#8	Description of trial design including type of trial (eg, parallel group, crossover, factorial, single group), allocation ratio, and framework (eg, superiority, equivalence, non-inferiority, exploratory)	9
Study setting	#9	Description of study settings (eg, community clinic, academic hospital) and list of countries where data will be collected. Reference to where list of study sites can be obtained	11, 17-18
Eligibility criteria	#10	Inclusion and exclusion criteria for participants. If applicable, eligibility criteria for study centres and individuals who will perform the interventions (eg, surgeons, psychotherapists)	9-10
Interventions: description	#11a	Interventions for each group with sufficient detail to allow replication, including how and when they will be administered	10-12

For peer review only

Interventions: modifications	#11b	Criteria for discontinuing or modifying allocated interventions for a given trial participant (eg, drug dose change in response to harms, participant request, or improving / worsening disease)	N/A
Interventions: adherance	#11c	Strategies to improve adherence to intervention protocols, and any procedures for monitoring adherence (eg, drug tablet return; laboratory tests)	12
Interventions: concomitant care	#11d	Relevant concomitant care and interventions that are permitted or prohibited during the trial	N/A
Outcomes	#12	Primary, secondary, and other outcomes, including the specific measurement variable (eg, systolic blood pressure), analysis metric (eg, change from baseline, final value, time to event), method of aggregation (eg, median, proportion), and time point for each outcome. Explanation of the clinical relevance of chosen efficacy and harm outcomes is strongly recommended	12-17
Participant timeline	#13	Time schedule of enrolment, interventions (including any run-ins and washouts), assessments, and visits for participants. A schematic diagram is highly recommended (see Figure)	17-18
Sample size	#14	Estimated number of participants needed to achieve study objectives and how it was determined, including clinical and statistical assumptions supporting any sample size calculations	12

For peer review only

Recruitment	#15	Strategies for achieving adequate participant enrolment to reach target sample size	17-18
Allocation: sequence generation	#16a	Method of generating the allocation sequence (eg, computer-generated random numbers), and list of any factors for stratification. To reduce predictability of a random sequence, details of any planned restriction (eg, blocking) should be provided in a separate document that is unavailable to those who enrol participants or assign interventions	9
Allocation concealment mechanism	#16b	Mechanism of implementing the allocation sequence (eg, central telephone; sequentially numbered, opaque, sealed envelopes), describing any steps to conceal the sequence until interventions are assigned	9, 18
Allocation: implementation	#16c	Who will generate the allocation sequence, who will enrol participants, and who will assign participants to interventions	9, 18
Blinding (masking)	#17a	Who will be blinded after assignment to interventions (eg, trial participants, care providers, outcome assessors, data analysts), and how	9, 18
Blinding (masking): emergency unblinding	#17b	If blinded, circumstances under which unblinding is permissible, and procedure for revealing a participant's allocated intervention during the trial	N/A

For peer review only

Data collection plan	#18a	Plans for assessment and collection of outcome, baseline, and other trial data, including any related processes to promote data quality (eg, duplicate measurements, training of assessors) and a description of study instruments (eg, questionnaires, laboratory tests) along with their reliability and validity, if known. Reference to where data collection forms can be found, if not in the protocol	12-18
Data collection plan: retention	#18b	Plans to promote participant retention and complete follow-up, including list of any outcome data to be collected for participants who discontinue or deviate from intervention protocols	18
Data management	#19	Plans for data entry, coding, security, and storage, including any related processes to promote data quality (eg, double data entry; range checks for data values). Reference to where details of data management procedures can be found, if not in the protocol	18-19
Statistics: outcomes	#20a	Statistical methods for analysing primary and secondary outcomes. Reference to where other details of the statistical analysis plan can be found, if not in the protocol	19-21
Statistics: additional analyses	#20b	Methods for any additional analyses (eg, subgroup and adjusted analyses)	N/A

For peer review only

Statistics: analysis population and missing data	#20c	Definition of analysis population relating to protocol non-adherence (eg, as randomised analysis), and any statistical methods to handle missing data (eg, multiple imputation)	19-20
Data monitoring: formal committee	#21a	Composition of data monitoring committee (DMC); summary of its role and reporting structure; statement of whether it is independent from the sponsor and competing interests; and reference to where further details about its charter can be found, if not in the protocol. Alternatively, an explanation of why a DMC is not needed	19, 22, 24
Data monitoring: interim analysis	#21b	Description of any interim analyses and stopping guidelines, including who will have access to these interim results and make the final decision to terminate the trial	N/A
Harms	#22	Plans for collecting, assessing, reporting, and managing solicited and spontaneously reported adverse events and other unintended effects of trial interventions or trial conduct	22
Auditing	#23	Frequency and procedures for auditing trial conduct, if any, and whether the process will be independent from investigators and the sponsor	N/A
Research ethics approval	#24	Plans for seeking research ethics committee / institutional review board (REC / IRB) approval	22

For peer review only

Protocol amendments	#25	Plans for communicating important protocol modifications (eg, changes to eligibility criteria, outcomes, analyses) to relevant parties (eg, investigators, REC / IRBs, trial participants, trial registries, journals, regulators)	24
Consent or assent	#26a	Who will obtain informed consent or assent from potential trial participants or authorised surrogates, and how (see Item 32)	22
Consent or assent: ancillary studies	#26b	Additional consent provisions for collection and use of participant data and biological specimens in ancillary studies, if applicable	N/A
Confidentiality	#27	How personal information about potential and enrolled participants will be collected, shared, and maintained in order to protect confidentiality before, during, and after the trial	19
Declaration of interests	#28	Financial and other competing interests for principal investigators for the overall trial and each study site	26
Data access	#29	Statement of who will have access to the final trial dataset, and disclosure of contractual agreements that limit such access for investigators	19
Ancillary and post trial care	#30	Provisions, if any, for ancillary and post-trial care, and for compensation to those who suffer harm from trial participation	N/A

For peer review only

Dissemination policy: trial results	#31a	Plans for investigators and sponsor to communicate trial results to participants, healthcare professionals, the public, and other relevant groups (eg, via publication, reporting in results databases, or other data sharing arrangements), including any publication restrictions	22-23
Dissemination policy: authorship	#31b	Authorship eligibility guidelines and any intended use of professional writers	N/A
Dissemination policy: reproducible research	#31c	Plans, if any, for granting public access to the full protocol, participant-level dataset, and statistical code	N/A
Informed consent materials	#32	Model consent form and other related documentation given to participants and authorised surrogates	On request from study team
Biological specimens	#33	Plans for collection, laboratory evaluation, and storage of biological specimens for genetic or molecular analysis in the current trial and for future use in ancillary studies, if applicable	N/A

The SPIRIT checklist is distributed under the terms of the Creative Commons Attribution License CC-BY-ND 3.0. This checklist can be completed online using https://www.goodreports.org/, a tool made by the EQUATOR Network in collaboration with Penelope.ai

For peer review only

BMJ Open

A novel group parenting intervention to reduce emotional and behavioural difficulties in young autistic children: Protocol for the Autism Spectrum Treatment and Resilience (ASTAR) pilot randomised controlled trial.

Journal:	*BMJ Open*
Manuscript ID	bmjopen-2019-029959.R1
Article Type:	Protocol
Date Submitted by the Author:	24-Apr-2019
Complete List of Authors:	Palmer, Melanie; Institute of Psychiatry, Psychology & Neuroscience, King's College London, Department of Child and Adolescent Psychiatry<br>Tarver, Joanne; Aston University School of Life and Health Sciences, Department of Psychology<br>Paris Perez, Juan; Institute of Psychiatry, Psychology & Neuroscience, King's College London, Department of Child and Adolescent Psychiatry<br>Cawthorne, Thomas; Institute of Psychiatry, Psychology & Neuroscience, King's College London, Department of Child and Adolescent Psychiatry<br>Romeo, Renee; Institute of Psychiatry, Psychology & Neuroscience, King's College London, Department of Health Service and Population Research<br>Stringer, Dominic; Institute of Psychiatry, Psychology & Neuroscience, King's College London, Department of Biostatistics and Health Informatics<br>Hallett, Victoria; South London and Maudsley NHS Foundation Trust<br>Mueller, Joanne; South London and Maudsley NHS Foundation Trust<br>Breese, Lauren; South London and Maudsley NHS Foundation Trust<br>Hollett, Megan; South London and Maudsley NHS Foundation Trust<br>Beresford, Bryony; University of York, Social Policy Research Unit<br>Knapp, Martin; London School of Economics, Personal Social Services Research Unit<br>Slonims , Vicky; Guy's and St Thomas' NHS Foundation Trust, Evelina Children's Hospital<br>Pickles, Andrew; Institute of Psychiatry, Psychology & Neuroscience, King's College London, Department of Biostatistics and Health Informatics<br>Simonoff, Emily; Institute of Psychiatry, Psychology & Neuroscience, King's College London, Department of Child and Adolescent Psychiatry; South London and Maudsley NHS Foundation Trust<br>Scott, Stephen; Institute of Psychiatry, Psychology & Neuroscience, King's College London, Department of Child and Adolescent Psychiatry; South London and Maudsley NHS Foundation Trust<br>Charman, Tony; King's College London, Institute of Psychiatry, Department of Psychology; South London and Maudsley NHS Foundation Trust
**Primary Subject Heading**:	Mental health

Secondary Subject Heading:	Evidence based practice, Health economics
Keywords:	Autism, Emotional and Behavioural Difficulties, Parenting Intervention, Feasibility, Pilot RCT

SCHOLARONE™
Manuscripts

TITLE: A novel group parenting intervention to reduce emotional and behavioural difficulties in young autistic children: Protocol for the Autism Spectrum Treatment and Resilience (ASTAR) pilot randomised controlled trial.

AUTHORS:

Melanie Palmer[1], Joanne Tarver[2,3], Juan Paris Perez[1], Thomas Cawthorne[1], Renee Romeo[1], Dominic Stringer[1], Victoria Hallett[4], Joanne Mueller[4], Lauren Breese[4], Megan Hollett[4], Bryony Beresford[5], Martin Knapp[6], Vicky Slonims[7], Andrew Pickles[1], Emily Simonoff[1,4], Stephen Scott[1,4] and Tony Charman[1,4].

AUTHOR AFFILIATIONS:

[1]King's College London, Institute of Psychiatry, Psychology & Neuroscience, London, UK.

[2]Department of Psychology, School of Life and Health Sciences, Aston University, Birmingham, UK

[3]Cerebra Centre for Neurodevelopmental Disorders, School of Psychology, University of Birmingham, Birmingham, UK

[4]South London and Maudsley NHS Foundation Trust, London, UK

[5]Social Policy Research Unit, University of York, York, UK

[6]Department of Health Policy, London School of Economics and Political Science, London, UK

[7]Newcomen Neurodevelopmental Centre, Children's Neurosciences, Evelina Children's Hospital, Guy's and St Thomas NHS Foundation Trust, London, UK.

For peer review only - http://bmjopen.bmj.com/site/about/guidelines.xhtml

CORRESPONDING AUTHOR:

The corresponding author, Melanie Palmer, can be contacted via email at melanie.palmer@kcl.ac.uk, or by telephone on +44 (0) 207 848 5260.

ORCID NUMBERS:

Melanie Palmer	0000-0001-5579-2170
Joanne Tarver	0000-0003-0555-6043
Juan Paris Perez	0000-0003-3171-0315
Thomas Cawthorne	0000-0003-4537-0016
Renee Romeo	0000-0003-3871-9697
Dominic Stringer	0000-0001-5624-1733
Victoria Hallett	0000-0002-7432-9824
Joanne Mueller	0000-0003-2737-1883
Lauren Breese	0000-0002-1246-7703
Megan Hollett	0000-0003-3123-1867
Bryony Beresford	0000-0003-0716-2902
Martin Knapp	0000-0003-1427-0215
Vicky Slonims	0000-0003-3339-2365
Andrew Pickles	0000-0003-1283-0346
Emily Simonoff	0000-0002-5450-0823
Stephen Scott	0000-0003-4680-6213
Tony Charman	0000-0003-1993-6549

For peer review only

ABSTRACT

Introduction: The majority of young autistic children display impairing emotional and behavioural difficulties that contribute to family stress. There is some evidence that behavioural parenting interventions are effective for reducing behavioural difficulties in autistic children, with less evidence assessing change in emotional difficulties. Previous trials have tended to use unblinded parent-report measures as primary outcomes and many do not employ an active control, limiting the conclusions that can be drawn.

Methods and analysis: The Autism Spectrum Treatment and Resilience (ASTAR) study is a pilot randomised controlled trial (RCT) testing the specific effect of a 12-week group parenting intervention (Predictive Parenting) on primary and secondary outcomes, in comparison to an attention control condition consisting of psychoeducation parent groups. Following a feasibility study to test research procedures and the interventions, the pilot RCT participants include 60 parents of 4-8 year old autistic children who are randomised to Predictive Parenting versus the attention control. Measures are administered at baseline and post-intervention to assess group differences in the child and parent outcomes, costs and service use, and adverse events. The primary outcome is an objective measure of child behaviour that challenges during interactions with their parent and a researcher. The trial aims to provide data on recruitment, retention, completion of measures and acceptability of the intervention and research protocol, in addition to providing a preliminary indication of potential efficacy and establishing an effect size that could be used to power a larger-scale efficacy trial. We will also provide preliminary estimates of the cost-effectiveness of the interventions.

Ethics and dissemination: Ethical approval was granted from NHS Camden and Kings Cross Research Ethics Committee (ref: 16/LO/1769) along with NHS R&D approval from South London and Maudsley, Guy's and St Thomas', and Croydon Health Services NHS

For peer review only - http://bmjopen.bmj.com/site/about/guidelines.xhtml

Trusts. The findings will be disseminated through publication in peer-reviewed journals and presentations at conferences.

Trial registration number: ISRCTN91411078.

Strengths and limitations of the study:

- The trial uses an objective measure as the primary outcome overcoming biases associated with participants being unblinded to treatment status.
- The target intervention, developed by clinicians with expertise in autism, is compared to an attention control condition to further guard against placebo effects.
- A feasibility study with nested qualitative evaluation enabled refinement of the intervention and research procedures prior to commencing the pilot RCT.
- Parents and autistic adults, referred to as patient and public involvement (PPI) panels, were involved in the development of the interventions and research procedures.
- As the study is a pilot RCT, conclusions about the efficacy of the intervention are not possible.

Keywords: Autism; Emotional and Behavioural Difficulties; Parenting Intervention; Feasibility; Pilot RCT.

For peer review only

INTRODUCTION

Background

Autism is characterised by difficulties in reciprocal social communication and the presence of restricted interests, repetitive behaviours and sensory anomalies.(1) At least 1% of children are autistic(2-4) and the condition is around three to four times more prevalent in males than females.(5) There are high rates of intellectual disability in autistic children with approximately 55% having an IQ below 70.(6) It has been demonstrated that additional psychiatric disorders frequently co-occur with autism at rates much higher than in the general population; up to 80-90% of young autistic children have additional emotional or behavioural difficulties meeting formal diagnostic criteria, with many having two or more additional disorders.(7-9) Anxiety disorders, attention deficit/hyperactivity disorder, and opposition defiant disorder are most common, and these difficulties tend to persist over time.(10)

Parents often report that it is these co-occurring difficulties, which are associated with poorer parental wellbeing and parental stress,(11) that they would like support with. Universal interventions are warranted given the high prevalence of co-occurring emotional and behavioural difficulties in autistic children. However, current service provision in the United Kingdom usually includes the offer of psychoeducation groups that focus on teaching parents about autism and developing strategies to support social and communication functioning, rather than the commonly co-occurring emotional and behavioural difficulties.

Behavioural parenting interventions are recommended by the National Institute of Health and Care Excellence(12) for the treatment of behavioural difficulties displayed by young children without autism. There are a number of effective parenting interventions that aim to reduce such difficulties in young autistic children. A recent meta-analysis of eight randomised controlled trials (RCTs) of behavioural parenting interventions aiming to reduce disruptive behaviour displayed by young autistic children(13) found a moderate effect on

For peer review only - http://bmjopen.bmj.com/site/about/guidelines.xhtml

disruptive behaviour when compared to controls (Standardised Mean Difference=-0.59, 95% confidence interval [CI] -0.88, -0.30). However, there was significant heterogeneity in the effect of parenting interventions on disruptive behaviour which may be due to sample size, mode of delivery and the focus and duration of treatment. Only one RCT included in the review involved anxiety management techniques even though anxiety disorders are the most common co-occurring psychiatric diagnoses in autism and "behaviour that challenges" is often described as an observable manifestation of anxiety.(14,15) A recent meta-analysis of 14 RCTs of cognitive behavioural therapy (CBT) interventions for anxiety in young autistic children, most of which included parental components, demonstrated that reductions in anxiety could be achieved.(16)

In addition, only one parenting intervention reviewed by Postorino et al.(13) included group-based sessions for parents, even though groups are more scalable and have the added benefit of providing a support network for parents. More than half of the included RCTs compared parenting interventions to a waitlist control or care as usual,(13) limiting conclusions that can be drawn about the effects as participants would not be blind to treatment allocation. Being unblinded to treatment allocation is particularly problematic when self-report measures are used as primary outcomes,(17) and there is a need for objective blinded measures of behaviour to be used as outcome measures in trials aiming to reduce emotional and behavioural difficulties displayed by young autistic children.

Aims and objectives

The Autism Spectrum Treatment and Resilience (ASTAR) trial is part of a research programme that aims to improve mental health outcomes among autistic individuals (Improving Autism Mental Health: https://iamhealthkcl.net/). ASTAR tests the specific effect of the Predictive Parenting intervention on child emotional and behavioural difficulties, in comparison to an attention control condition (psychoeducation parent groups). The aims of

For peer review only - http://bmjopen.bmj.com/site/about/guidelines.xhtml

the ASTAR trial are to: (1) examine the feasibility of the intervention in terms of recruitment, retention, completion of research measures and acceptability to parents; (2) provide a preliminary indication of potential efficacy on the primary and secondary outcomes and establish an effect size that could be used to power a future larger scale RCT; and (3) provide preliminary estimates of the cost-effectiveness of the intervention to inform a larger trial.

Consistent with Medical Research Council guidance on evaluating complex interventions,(18) we first conducted a preliminary feasibility phase testing the proposed research procedures and the Predictive Parenting (target intervention) and psychoeducation (control) group interventions with families with a 4-8 year old autistic child. A nested qualitative evaluation was conducted to explore the views of parents who declined to take part, those who completed/dropped-out of the interventions and the group facilitators. Findings from the feasibility phase were used to amend the research procedures and intervention manuals prior to the subsequent pilot RCT (see below for further information on learning from the feasibility phase).

The primary outcome of the pilot RCT is observed child behaviour that challenges, captured during a structured researcher- and parent-child interaction assessment (see description of measure below for further details). Secondary outcomes are child compliance and child-centred and child-directive parenting captured from the same observation and parent- and teacher-report of child emotional and behavioural difficulties. We are also measuring the effects of the interventions on parental stress and wellbeing, parenting practices and parenting self-efficacy.

METHODS AND ANALYSIS

Learning from the feasibility phase

The aim of the feasibility phase was to test the proposed recruitment processes and rates, the adequacy and acceptability of proposed measures and obtain the views of parents

For peer review only - http://bmjopen.bmj.com/site/about/guidelines.xhtml

and professionals on the research processes and interventions. Participants were 22 families (91% mothers and 9% fathers) with a 4-8 year old child with a clinical diagnosis of autism spectrum disorder. All but one of the children were male, and children were spilt across mainstream (*n*=10) and two special schools (*n*=12). Children in the special schools groups attended either a mixed autism-specific special school or a special school catering for children with severe learning difficulties co-occurring with autism. As intervention content is differentiated by child verbal ability, parents of minimally verbal children (*n*=12) attended groups separately from parents of verbal children (*n*=10).

We recruited 22 out of our target of 24 (92%) for the feasibility phase and we retained 20/22 (91%) families in the research protocol to post-intervention, indicating that the research processes were acceptable to families. All 22 parents gave consent for their child's teacher to complete measures. Baseline teacher questionnaires were obtained for 20/22 (91%) children and retention of teachers at post-intervention was high (18/22, 82%).

Parents who were interviewed reported that the research procedures were acceptable, although some felt the assessment process was lengthy. Prior to commencing the pilot RCT, two proposed outcome measures were removed to reduce burden on families (see our ISRCTN record for a log of outcome measures tested during the feasibility phase). For some parents, there appeared to have been a lack of clarity about the difference between the research and clinical teams and who they would have contact with at each stage of the study. This led to amendments in the information given to parents to help make this distinction clearer. Findings from the qualitative interviews indicated that most parents reported that they found the groups helpful and that they enjoyed meeting other parents in a similar situation. Feedback on the structure, timing, course materials and homework led to modifications to the Predictive Parenting intervention. For example, changes were made to make the groups more accessible and relevant to parents of children with lower levels of verbal ability. The study

For peer review only - http://bmjopen.bmj.com/site/about/guidelines.xhtml

design was also amended by increasing the number of families in each group (from six to eight) as it was a more efficient way to recruit and deliver the interventions. The increased group size was not thought to disrupt the intervention; indeed the slightly larger sizes may be helpful for group dynamics. Further details on the feasibility study can be provided upon contact with the research team.

Patient and Public Involvement (PPI)

Panels of parents of autistic children and autistic adults have been involved in all phases of the study and assisted with the development of the intervention curriculums and adaptions for parents of minimally verbal children, as well as advising on the research procedures. Guidance and advice about language to use when speaking with parents about the therapy goals and research processes (including on the written materials such as flyers and information sheets) was given.

Trial design

The study is a parallel group pilot RCT. Participating families are allocated to one of two treatment arms (Predictive Parenting or psychoeducational parent groups). Randomisation is conducted on blocks of 10-18 families on a ratio of 1:1, resulting in groups of 5-9 families in each treatment arm for any block. The randomisation algorithm is run by an independent statistician within the Biostatistics and Health Informatics Department, IoPPN, King's College London. Details of this are recorded in a separate randomisation specification document. Intervention allocation is emailed only to the group facilitators to ensure that the researchers are blind to condition.

Measures are collected at baseline, up to 2 months prior to the planned randomisation date, and approximately 18-24 weeks after randomisation once the 12-week intervention has finished. Group differences in outcomes will be examined.

Inclusion criteria

For peer review only - http://bmjopen.bmj.com/site/about/guidelines.xhtml

- Parent/carer of an autistic child, as confirmed by their clinician, aged between 4:0 years and 8:11 years
- Have sufficient spoken English to access the intervention
- Agree that their family doctor can be informed of their involvement in the trial.

Exclusion criteria

- Current participation in a behavioural parenting intervention delivered by another service
- Child has epileptic seizures more than weekly
- Parent or child has a severe hearing or visual impairment
- Active significant safeguarding concerns or a current severe parental psychiatric disorder
- Participation in the initial feasibility phase.

Interventions

Predictive Parenting (target intervention)

Predictive Parenting builds on behavioural parenting interventions, an evidence-based, well-accepted and cost-effective approach to targeting disruptive behaviour in children without autism.(12) It also incorporates well-established parent-mediated cognitive-behavioural therapy strategies for managing child anxiety.(16) It consists of 12 weekly 2-hour groups which extend parents' understanding of autism and associated difficulties and focus on supporting parents to understand and manage their child's emotions and behaviours (see Table 1 for content covered in Predictive Parenting). Techniques for helping parents prevent and reduce disruptive behaviour and anxiety are taught. It also includes content on promoting parental self-care and stress reduction. Content is adapted based on child verbal ability (minimally verbal vs. verbal). In addition to the 12 group sessions, two individual sessions are conducted- one between sessions 2 and 4 and the other between sessions 10 and

For peer review only - http://bmjopen.bmj.com/site/about/guidelines.xhtml

For peer review only

12. These individual sessions are up to 60 minutes long and aim to support individualisation and generalisation of the strategies for each family. The intervention is conducted in the community in local child and adolescent mental health services, libraries, or schools. Further information about Predictive Parenting will be published in a separate manuscript.

Table 1. Table displaying the content covered in Predictive Parenting

**Group session**	**Content**
1	Understanding ASD
2	Becoming a Behaviour Predictor
3	The Power of Planning
4	Predictably Positive Household
5	Clever Communication
6	Predictable Praise and Rewards
7	Managing Challenging Behaviour and Meltdowns
8	Predictable Parent Action Plans
9	Understanding Anxiety
10	Anxiety and Unpredictability Toolkit 1
11	Anxiety and Unpredictability Toolkit 2
12	Looking Forward and Looking After Yourself

Psychoeducational parent group (attention control condition)

The 'Seven Cs of ASD', the attention control condition, also consists of 12 weekly 2-hour groups that aim to provide psychoeducation and social support, whilst not providing specific guidance on managing behaviours or emotions. Table 2 below displays the content covered in each session of The Seven Cs of ASD. Like Predictive Parenting, content is adapted based on child verbal ability.

Table 2. Table displaying the content covered in The Seven Cs of ASD

**Group session**	**Content**
1	Introduction and understanding ASD
2	Causes of ASD
3	Concepts in ASD
4	Caring for yourself and your family: Part 1
5	Caring for yourself and your family: Part 2
6	Co-morbidities in ASD: Part 1
7	Co-morbidities in ASD: Part 2
8	Clinical treatments for ASD
9	Communication and advocating for your child

For peer review only - http://bmjopen.bmj.com/site/about/guidelines.xhtml

10	Classroom considerations
11	Caring for yourself and your family: Part 3
12	Recap and review

Intervention adherence

Detailed intervention manuals have been developed and frequent clinical supervision is provided to reduce variability due to therapist effects. Checklists have been developed to measure intervention fidelity, which assess session content and group process. These are completed by the group facilitators after each intervention session.

Sample size justification

As this is a pilot RCT, a formal sample size calculation was not undertaken. We are recruiting 60 families into the pilot RCT. We expect that retention will be approximately 90%, as reported by other trials of psychological intervention conducted with parents of young autistic children. We expect a more modest effect size than the 1.3 reported by Sofronoff et al.(19) as this was for a parent-reported measure and therefore unblinded. For the comparison of Predictive Parenting and the attention control condition, power was calculated by a non-central chi-square method using a linear mixed model with baseline (baseline-outcome correlation assumed 0.7) as covariate for two-tailed p=.05 and intraclass correlation for within intervention group of 0.02 and 10% drop-out. For an effect size (ES) of 0.5, our study has an expected 95% CI of 0.08, 0.92 and power of 64%, while for an ES of 0.6 the expected 95% CI is 0.18, 1.02 and 79% power.

Outcomes

Table 3 below displays measures that are being used in the trial and when they are administered.

Primary outcome

The primary outcome measure is child behaviour that challenges displayed during an observation of researcher-child and parent-child interactions. We have developed the

For peer review only - http://bmjopen.bmj.com/site/about/guidelines.xhtml

Observation Schedule for Children with Autism – Anxiety and Behaviour (OSCA–AB) for the trial drawing on existing well-validated observational measures of parent-child interaction.(20-23) Two researcher-led and six parent-led tasks are completed during the 20-25 minute observation. Tasks aim to simulate everyday challenges that autistic children may face and find difficult. The frequency of a range of child behaviour that challenges (destructive behaviour, aggression towards themselves and others, frustrated vocalisations, non-compliance, avoidance and reassurance seeking) observed during the OSCA–AB are coded. As the length of the observation varies, the rate of child behaviour that challenges per minute is calculated. Further information about the measure will be published in a separate manuscript.

Secondary outcomes

Observed child compliance

The frequency of observed child compliance during the OSCA–AB is coded and the rate of child compliance per minute is calculated.

Observed parent behaviour

Frequencies of a range of observed parent behaviour (e.g., positive and negative comments, commands, giving the child opportunity to comply, praise, physical handling and supportive physical guidance) during the OSCA-AB are coded and differences between groups will be examined. Child-centred parenting behaviours (positive comments, clear commands, praise and supportive physical guidance) and child-directive parenting behaviours (negative comments, unclear commands, no opportunity to comply and physical handling) are summed to produce total child-centred parenting behaviour and child-directive parenting behaviour scores. Due to variation in the length of the observation, rates of child-centred and child-directive parenting behaviours per minute are calculated. The proportion of child-

For peer review only - http://bmjopen.bmj.com/site/about/guidelines.xhtml

centred parenting behaviour / child-centred and child-directive parenting behaviours is also calculated.

Parent-reported child emotional and behavioural difficulties

Parent-rated child emotional and behavioural difficulties is measured using The Aberrant Behaviour Checklist (ABC)(24) Irritability and Hyperactivity subscales. The Assessment of Concerning Behaviours (ACB) scale,(25) a measure of child mental health and concerning behaviours developed specifically for use with autistic individuals, is also completed. Forty-four items are rated on a 5-point sliding scale anchored by opposing responses ('not at all' and 'very much'). The Home Situations Questionnaire-Autism Spectrum Disorders (HSQ-ASD),(26) an autism-specific measure of child non-compliance in everyday situations is also administered. Parent-reported child anxiety is measured using the Preschool Anxiety Scale Revised (PASR),(27) which taps into specific fears, and generalised, social and separation anxiety.

A narrative describing one or two of the most pressing problems for parents related to child emotions and behaviours (Parent-Nominated Target Problems) is elicited at baseline. Information on the presentation, frequency, duration, intensity and interference with daily function, family life and other consequences is sought.(28) The narratives are reviewed at post-intervention and change from baseline is scored on a 9-point scale. The Clinical Global Impression-Improvement (CGI-I)(29) is used to rate overall improvement in child emotional and behavioural difficulties based on the parent-nominated target problems and parental perceptions of improvement.

Teacher-reported child emotional and behavioural difficulties

The ABC(24) Irritability and Hyperactivity subscales is completed by the child's teacher or someone involved in their education (e.g., key worker, Special Educational Needs Co-ordinator). The teacher version of the ACB(25) is also completed.

For peer review only - http://bmjopen.bmj.com/site/about/guidelines.xhtml

Parent-reported parenting outcomes

Parent-rated parenting stress associated with core and co-morbid symptoms is measured using the Autism Parenting Stress Index (APSI)(30) and parenting self-efficacy is measured using the Child Adjustment and Parent Efficacy Scale-Developmental Disability (CAPES-DD) Parent Efficacy subscale,(31) a 16-item scale assessing confidence in managing specific child behaviours. The Short Warwick-Edinburgh Mental Wellbeing Scale (SWEMWBS)(32) assesses parent reports of their own wellbeing. The short version of the Parenting Scale (PS)(33) is used to measure self-reported lax and overreactive parenting practices.

Sample characterisation measures

Demographic information about the family is obtained at baseline. Autism severity is measured at baseline only using the parent-reported Social Communication Questionnaire-Lifetime version (SCQ-L)(34), along with the ADOS–2.(35) The ADOS–2 is the gold standard observation for assessing autism symptoms and is administered by trained researchers. The Adaptive Behaviour Assessment System – 3rd edition (ABAS–3)(36) is completed by parents at baseline and measures three broad domains of adaptive skills and functioning (conceptual, social and practical), resulting in a General Adaptive Composite score.

Intervention related measures

Attendance at intervention sessions and retention in the intervention is recorded. Satisfaction with the content and delivery of both interventions is measured using questionnaires developed for study.

Health economic measures

Parental wellbeing and daily emotions are measured using the Office of National Statistics (ONS) Personal Wellbeing questions(37) which ask about life satisfaction, worth,

For peer review only - http://bmjopen.bmj.com/site/about/guidelines.xhtml

happiness, and anxiety. The EQ-5D-5L(38) is used to measure parent reports of their own health-related quality of life, and index-based values are available to enable quality-adjusted life years (QALYs) calculations to be used in the cost-effectiveness analysis.

An adapted version of the Client Service Receipt Inventory (CSRI)(39) measures service use and cost-related impacts at baseline and post-intervention, to inform the cost-effectiveness analysis. Parents are asked to retrospectively identify all public, private and voluntary sector services used by the child, as well as services used by other family members that are linked to the child's autism or emotional and behavioural difficulties. The CSRI also includes information on unpaid support and employment impacts on other family members. The facilitators delivering the interventions track their time spent on intervention-related activities and travel costs to be used in costing the interventions.

For peer review only

Table 3. Table showing administration of measures.

Measure	Baseline	During treatment	Post-intervention	Completed by
Primary outcome				
OSCA–AB Child Behaviour That Challenges	✓		✓	Blinded researcher
Secondary outcomes				
OSCA–AB Child Compliance	✓		✓	Blinded researcher
OSCA–AB Child-Centred Parenting Behaviour	✓		✓	Blinded researcher
OSCA–AB Child-Directive Parenting Behaviour	✓		✓	Blinded researcher
ABC Irritability and Hyperactivity	✓		✓	Parent/teacher
ACB	✓		✓	Parent/teacher
HSQ-ASD	✓		✓	Parent
PASR	✓		✓	Parent
Improvement in Parent-Nominated Target Problems	✓		✓	Parent/blinded researcher
CGI-I	✓		✓	Parent/blinded researcher
APSI	✓		✓	Parent

CAPES-DD Parent Efficacy	✓		✓	Parent
SWEMWBS	✓		✓	Parent
PS	✓		✓	Parent
Adverse events			✓	Parent/blinded researcher
Sample characterisation				
Demographics	✓			Parent
SCQ-Lifetime	✓			Parent
ADOS-2	✓			Blinded researcher
ABAS-3	✓			Parent
Intervention related measures				
Intervention attendance		✓		Clinician
Intervention satisfaction			✓	Parent
Intervention fidelity		✓		Clinician
Health economics measures				
ONS Personal Wellbeing	✓		✓	Parent
EQ-5D-5L Quality of Life	✓		✓	Parent
CSRI	✓		✓	Parent/blinded researcher
Facilitator time use		✓		Clinician

Note.
ABAS–3=Adaptive Behaviour Assessment System – 3rd edition; ABC=Aberrant Behaviour Checklist; ACB=Assessment of Concerning Behaviour; ADOS–2=Autism Diagnostic Observation Schedule – 2nd edition; APSI=Autism Parenting Stress Index; CAPES-DD=Child Adjustment and Parent Efficacy Scale-Developmental Disability; CGI-I=Clinical Global Impression-Improvement; CSRI= Client Service Receipt Inventory; HSQ-ASD=Home Situations Questionnaire-Autism Spectrum Disorders; ONS=Office of National Statistics; OSCA–AB=Observation Schedule for Children with Autism – Anxiety and Behaviour; PASR= Preschool Anxiety Scale Revised; PS= Parenting Scale; SWEMWBS=Short Warwick-Edinburgh Mental Wellbeing Scale; SCQ=Social Communication Questionnaire.

Procedure

Children between the ages of 4 and 8 years with a diagnosis of autism spectrum disorder (ASD) are recruited to the study from participating services following referral via local autism diagnostic teams, education professionals, support groups and consented databases. Potential participants can also self-refer. As the intervention content is adapted based on child verbal ability, the groups are run separately with parents of minimally verbal

For peer review only - http://bmjopen.bmj.com/site/about/guidelines.xhtml

and verbal children within each of our localities. Therefore, the blocks of 10-18 families recruited for allocation to condition will be stratified by verbal ability level (minimally verbal [defined as Autism Diagnostic Observation Schedule – 2nd edition, ADOS–2(35) Module 1] vs. verbal children [defined as ADOS–2 Module 2 or above]) and by locality (Croydon, Bromley) as part of the recruitment procedure.

After initial contact and pre-screening for eligibility, research staff obtain informed consent and conduct baseline assessments to confirm eligibility. All families are assigned a unique participant ID. Questionnaire measures are completed online or in hard copy depending on the parent's preference. Other measures are completed during a visit to the research setting, over the phone or at the child's school. Baseline assessments with families are conducted up to 2 months prior to randomisation. With parental consent, teachers are asked to complete questionnaires about the child's emotional and behavioural difficulties at school. Post-intervention assessments are conducted after the completion of the intervention. Outcome measures are sought for all families regardless of their participation in the treatment provided.

There are separate research and clinical teams who are based in different buildings and have separate supervision structures. The assessments and interventions are conducted in a way to avoid inadvertent divulging of information that could reveal allocation status. The location and materials used during the research assessments are different in type and location to those used for the intervention sessions, avoiding any familiarity effect for parents. Researchers involved in conducting the assessments and rating outcome measures are blind to intervention content and participant condition. Group facilitators are blind to primary outcome measurement.

Data management, confidentiality and access

For peer review only - http://bmjopen.bmj.com/site/about/guidelines.xhtml

All data in the trial are anonymised. All paper records are filed anonymously by the participant's unique study number in secure locked cabinets in the Department of Child and Adolescent Psychiatry, IoPPN, King's College London. Consent forms are stored separately. Personal details (e.g., name, address, telephone numbers) are stored in a separate encrypted database and linked by initial, date of birth and unique participant ID number. Some records from the feasibility phase are stored securely at York University.

Data from paper case report forms are entered on SPSS databases and along with other electronic data, stored on a King's server folder that is only accessible to the research team. Double data entry will be completed on at least 10% of all entered data and quality checks will be conducted. The principal investigator, trial statisticians and other members of the study team have access to final datasets and will undertake analysis as appropriate and necessary. Any arrangements for other researchers to have access to the data will be negotiated separately and the Central Office of Research Ethics Committee will be informed.

Statistical analyses

A statistical analysis plan has been written by the trial statisticians (AP and DS) and will be approved by the chief investigator, the Trial Steering Committee (TSC) and the Data Monitoring Committee (DMC) prior to any analysis being undertaken. The analyses will be carried out using Stata.

In accordance with CONSORT guidelines, we will report the flow of participants through the trial. Descriptive statistics of recruitment, drop-out and completeness of assessments and interventions will be provided. Satisfaction and fidelity of the intervention will also be reported descriptively. Baseline characteristics will be presented by group.

The main analysis will be via intention-to-treat, including all participants who were randomised. It will use statistical techniques for handling missing outcome data under a missing at random assumption and multiple imputation for missing measures will be

For peer review only - http://bmjopen.bmj.com/site/about/guidelines.xhtml

considered. We will test for a between-group change in the primary outcome at post-intervention, using ANCOVA regression predicting outcome where post-intervention is also covaried for baseline. Dummy variables will be used to account for randomisation stratification and the clustering effects of groups. The distribution of the primary outcome at baseline will be examined for evidence of floor effects. Where floor effects are present, a generalised mixed model/structural equation modelling setup, in which both baseline and post-intervention are modelled as potentially censored response variables, will be used with a covariance between equations that yields the ANCOVA estimate of treatment effect in the absence of censoring. Secondary outcome measures will be analysed in the same way. Analysis of all post-intervention treatment effects will be undertaken after all post-intervention outcome measures are completed. Trial statisticians will remain blind until after the primary and secondary outcomes are analysed.

Economic evaluation

The cost for each participant in the pilot will be derived by the product of the quantity of each service and support used and the unit cost of each of them. Unit costs will be based on the economic notion of opportunity costs – which considers the value of the resource in its next best alternative use. Where this is not practicable, unit costs will be approximated by nationally representative health and personal social services tariffs. Where unit costs are not readily available from such sources, we will derive costs using approaches outlined in an annual compendium of Unit Cost of Health and Social Care. We will use the most recent publication of the *Unit Cost of Health and Social Care* produced by the Personal Social Services Research Unit at the time of analysis. All other reported costs will be consistent with the price level used in that edition.(40)

When applying unit costs to unpaid care, we will use other approaches such as replacement costs. Under this approach, unpaid care by family and other carers will be costed

For peer review only - http://bmjopen.bmj.com/site/about/guidelines.xhtml

using the average hourly rate for a local authority home care worker as the assumed cost for each hour of unpaid informal care.

Consistent with the outcome analyses, the economic evaluation will also conduct an intention-to-treat analysis, including all participants who were randomised. We will compute and compare comprehensively measured costs (for each of the two perspectives adopted: health and social care, public sector or societal) for the two interventions. Under each perspective, the cost-effectiveness analyses will bring together costs and the primary outcome and will compute indicative incremental cost-effectiveness ratios and net benefits; the societal perspective will be adopted in the main analyses. In a secondary economic evaluation, QALY gains computed from parental EQ-5D-5L scores will be compared with costs from each perspective; again, the societal perspective will be adopted to facilitate comparisons with the main analyses. Other exploratory cost-effectiveness analyses will examine other outcomes and perspectives.

In each case, an incremental cost-effectiveness ratio will be computed as the mean cost difference between Predictive Parenting and the attention control condition divided by the mean difference in change in measures of outcome respectively. If one treatment is indicating it is likely to be both more effective and costlier than the other, we would consider if there is some suggestion that it is worth incurring the higher costs in order to achieve the improved outcomes. The approach we will employ to reveal the nature of trade-offs such as these – and to represent the inherent uncertainty in any evaluation – will be to plot cost-effectiveness acceptability curves generated from bootstrap analyses. Sensitivity analyses will explore the impact of key assumptions such as the costing of unpaid care time and lost productivity, and the choice of outcome.

ETHICS AND DISSEMINATION

For peer review only - http://bmjopen.bmj.com/site/about/guidelines.xhtml

Ethical approval was granted from NHS Camden and Kings Cross Research Ethics Committee on 18/11/2016 (ref: 16/LO/1769). Written consent is obtained from all participating parents. Assent from children is obtained where appropriate. The SPIRIT reporting guidelines are followed for this protocol.(41)

For the pilot RCT, we formed a TSC which includes an independent chair, independent members and parent representatives (see below for membership). The TSC met prior to the commencement of the pilot RCT to agree the study protocol and will meet at least annually thereafter. The TSC were consulted on the study protocol, techniques for ascertainment and the focus of measurement including the primary outcome. They were also consulted on whether a DMC is required and decided that a sub-committee of the TSC (consisting of the chair and statistician) could act as the DMC.

Adverse events are measured at post-intervention and include events related to child, parent and family wellbeing that may not be captured by outcome measures (e.g., increased family discord, school refusal, significant change in a sibling's wellbeing or behaviour) as well as pre-defined standard medical events. Such events that arise during treatment are documented when a situation becomes known to group facilitators. The TSC and DMC have independent oversight of the study and are informed of all adverse events.

This trial will contribute to the literature on parenting interventions for reducing emotional and behavioural difficulties displayed by young autistic children. As the study is a pilot RCT, conclusions about the efficacy of the intervention are not possible. However, the study design enables us to consider the feasibility of conducting a large-scale RCT to test the efficacy of Predictive Parenting. The findings from the pilot RCT will be disseminated through publication in peer-reviewed journals of general and special interest and presentations at national and international conferences. There will also be a general

For peer review only - http://bmjopen.bmj.com/site/about/guidelines.xhtml

dissemination programme for families including participants co-ordinated through our collaborators in the National Autistic Society.

For peer review only

For peer review only - http://bmjopen.bmj.com/site/about/guidelines.xhtml

TRIAL STATUS

Protocol version 1.4, dated 04/02/2019, see our ISRCTN record for log of protocol amendments. Recruitment was completed on the 16/10/2018. Post-intervention assessments are due for completion by 30/04/2019.

TRIAL SPONSOR

King's College London and South London and Maudsley NHS Foundation Trust. Email: slam-ioppn.research@kcl.ac.uk.

TRIAL STEERING COMMITTEE

Professor Alan Stein, University of Oxford (Chair); Dr Matt Sydes, MRC Clinical Trials Unit, University College London (Member); Dr Jacqueline Rodgers, University of Newcastle (Member); Bridget Gilchrist (Parent Representative); Lindsay Stairs (Parent Representative).

DATA MONITORING COMMITTEE

As the trial is a pilot RCT, the TSC agreed that a subgroup consisting of Professor Alan Stein and Dr Matt Sydes would act as the DMC for ASTAR.

ACKNOWLEDGEMENTS

We are grateful to the families who were involved in the initial feasibility study and to those who are involved in the pilot trial. We would like to thank other members of the IAMHealth consortium for their comments and advice on the trial design. We would also like to thank local professionals who assisted with identifying potential participants, with particular thanks to Dr Shade Alu, Sarran Bond, Marion Drennan, Dr Mark O'Leary, Dr Fernando Salazar and Jackie Sutherland. We would also like to acknowledge Katherine Appleby, Elena Baker,

For peer review only

Emma Biggin, Sophie Carruthers, Margot Frayne, Lydia Johnson-Ferguson, Moriya Maccabee and Sophie Webb for their assistance with data collection.

AUTHORS' CONTRIBUTIONS

MP, JT, JPP, RR, DS, BB, MK, VS, AP, ES, SS and TC were involved in designing the study and drafting the protocol for the pilot RCT. TCa is involved in recruiting and collecting data for the pilot RCT. VH, JM, LB and MH are involved in developing and delivering the interventions. The manuscript was drafted by MP and all authors read, made revisions and approved the final version.

FUNDING STATEMENT

This trial summarises independent research funded by the National Institute for Health Research (NIHR) under its Programme Grants for Applied Research programme (RP-PG-1211-20016). The views expressed in this presentation are those of the authors and not necessarily those of the NHS, the NIHR or the Department of Health. Additional funding for intervention materials was received from the Maudsley Charity (1157). MK receives support from the NIHR School for Social Care Research. AP receives support from the NIHR through a Senior Investigator Award (NF-SI-0617-10120). ES additionally receives support from the National Institute for Health Research (NIHR) Biomedical Research Centre at South London and Maudsley Foundation Trust (IS-BRC-1215-20018), the NIHR through a Senior Investigator Award (NF-SI-0514-10073), the European Union Innovative Medicines Initiative (EU-IMI 115300), Autistica (7237), Medical Research Council (MR/R000832/1, MR/P019293/1), the Economic and Social Research Council (ESRC 003041/1), Guy's and St Thomas' Charitable Foundation (GSTT EF1150502) and the Maudsley Charity. TC receives

For peer review only - http://bmjopen.bmj.com/site/about/guidelines.xhtml

grant or research support from the NIHR, the Medical Research Council, the European Union (IMI, H2020), Autistica, MQ and The Waterloo Foundation.

COMPETING INTERESTS

AP declares that he receives royalties from WPS for the Social Communication Questionnaire.

PATIENT CONSENT

Obtained.

ETHICAL APPROVAL

Obtained from NHS Camden and Kings Cross Research Ethics Committee (ref: 16/LO/1769).

PROVENANCE AND PEER REVIEW

Not commissioned; externally peer reviewed for funding and subsequently ethical approval prior to submission.

For peer review only

For peer review only - http://bmjopen.bmj.com/site/about/guidelines.xhtml

REFERENCES

(1) American Psychiatric Association. Diagnostic and Statistical Manual of Mental Disorders: DSM-5 (5th ed.). Arlington, VA: American Psychiatric Association 2013.

(2) Baird G, Simonoff E, Pickles A, et al. Prevalence of disorders of the autism spectrum in a population cohort of children in South Thames: the Special Needs and Autism Project (SNAP). *Lancet* 2006;368:210–215.

(3) Baio J, Wiggins L, Christensen DL, et al. Prevalence of autism spectrum disorder among children aged 8 years - Autism and Developmental Disabilities Monitoring Network, 11 Sites, United States, 2014. *MMWR Surveill* 2018;67:1–23.

(4) Russell G, Rodgers LR, Ukoumunne OC, et al. Prevalence of parent-reported ASD and ADHD in the UK: Findings from the Millennium Cohort Study. *J Autism Dev Disord* 2014;44:31–40.

(5) Loomes R, Hull L, Mandy WPL. What is the male-to-female ratio in autism spectrum disorder? A systematic review and meta-analysis. *J Am Acad Child Adolesc Psychiatry* 2017 ;56:466–474.

(6) Charman T, Pickles A, Simonoff E, et al. IQ in children with autism spectrum disorders: data from the Special Needs and Autism Project (SNAP). *Psychol Med* 2011;41:619–627.

(7) Salazar F, Baird G, Chandler S, et al. Co-occurring psychiatric disorders in preschool and elementary school-aged children with autism spectrum disorder. *J Autism Dev Disord* 2015;40:1080–1093.

(8) Chandler S, Howlin P, Simonoff E, et al. Emotional and behavioural problems in young children with autism spectrum disorder. *Dev Med Child Neurol* 2016;58:202–208.

(9) Simonoff E, Pickles A, Charman T, et al. Psychiatric disorders in children with autism spectrum disorders: Prevalence, comorbidity, and associated factors in a population-derived sample. *J Am Acad Child Adolesc Psychiatry* 2008;47:921–929.

(10) Simonoff E, Jones C R, Baird G, et al. The persistence and stability of psychiatric problems in adolescents with autism spectrum disorders. *J Child Psychol Psychiatry* 2013;54:186–194.

(11) Yorke I, White P, Weston A, et al. The association between emotional and behavioral problems in children with autism spectrum disorder and psychological distress in their parents: A systematic review and meta-analysis. *J Autism Dev Disord* 2018;48:3393–3415.

(12) National Institute for Health and Clinical Excellence. Antisocial behaviour and conduct disorders in children and young people: Recognition and management (CG158). 2013. https://www.nice.org.uk/guidance/cg158

For peer review only

For peer review only - http://bmjopen.bmj.com/site/about/guidelines.xhtml

(13) Postorino V, Sharp WG, McCracken CE, et al. A systematic review and meta-analysis of parent training for disruptive behavior in children with autism spectrum disorder. *Clin Child Fam Psychol Rev* 2017;20:391–402.

(14) Ozsivadjian A, Knott FF, Magiati I. Parent and child perspectives on the nature of anxiety in children and young people with autism spectrum disorders: A focus group study. *Autism* 2012;16:107–121.

(15) Bearss K, Taylor CA, Aman MG, et al. Using qualitative methods to guide scale development for anxiety in youth with autism spectrum disorder. *Autism* 2015;20:663–672.

(16) Ung D, Selles R, Small BJ, et al. A systematic review and meta-analysis of cognitive-behavioral therapy for anxiety in youth with high-functioning autism spectrum disorders. *Child Psychiatry Hum Dev* 2015;46:533–547.

(17) Sonuga-Barke EJ, Brandeis D, Cortese S, et al. Nonpharmacological interventions for ADHD: Systematic review and meta-analyses of randomized controlled trials of dietary and psychological treatments. *Am J Psychiatry* 2013;170:275–289.

(18) Craig P, Dieppe P, Macintyre S, et al. Developing and evaluating complex interventions: The new Medical Research Council guidance. *BMJ* 2008;337:a1655.

(19) Sofronoff K, Attwood T, Hinton S. A randomised controlled trial of a CBT intervention for anxiety in children with asperger syndrome. *J Child Psychol Psychiatry* 2005;46:1152–1160.

(20) Wakschlag LS, Hill C, Carter AS, et al. Observational assessment of preschool disruptive behavior, part I: Reliability of the Disruptive Behavior Diagnostic Observation Schedule (DB-DOS). *J Am Acad Child Adolesc Psychiatry* 2008;47:622–631.

(21) Robinson EA, Eyberg SM. The dyadic parent-child interaction coding system: Standardization and validation. *J Consult Clin Psychol* 1981;49:245–250.

(22) Johnson CR, Butter EM, Handen BL et al. Standardised Observation Analogue Procedure (SOAP) for assessing parent and child behaviours in clinical trials. *J Intellect Dev Disabil* 2009;34:230–238.

(23) Mian ND, Carter AS, Pine DS, et al. Development of a novel observational measure for anxiety in young children: The Anxiety Dimensional Observation Scale. *J Child Psychol Psychiatry* 2015;56:1017–1025.

(24) Aman MG, Singh NN, Stewart AW, et al. The Aberrant Behavior Checklist: A behavior rating scale for the assessment of treatment effects. *Am J Ment Defic* 1985;89:485–491.

(25) Santosh P, Tarver J, Gibbons F, et al. Protocol for the development and validation of a questionnaire to assess concerning behaviours and mental health in individuals with autism spectrum disorders: The Assessment of Concerning Behaviour (ACB) scale. *BMJ Open* 2016;6:e010693.

For peer review only - http://bmjopen.bmj.com/site/about/guidelines.xhtml

(26) Chowdhury M, Aman MG, Lecavalier L, et al. Factor structure and psychometric properties of the revised Home Situations Questionnaire for autism spectrum disorder: The Home Situations Questionnaire-Autism Spectrum Disorder. *Autism* 2015;54:281–291.

(27) Edwards SL, Rapee RM, Kennedy SJ, et al. The assessment of anxiety symptoms in preschool-aged children: The revised Preschool Anxiety Scale. *J Clin Child Adolesc Psychol* 2010;39:400–409.

(28) Arnold LE, Vitiello B, McDougle C, et al. Parent-defined target symptoms respond to risperidone in RUPP autism study: Customer approach to clinical trials. *J Am Acad Child Adolesc Psychiatry* 2003;42:1443-1450.

(29) Guy W. ECDEU Assessment Manual for Psychopharmacology: Publication ADM 76-338. 1976.

(30) Silva LM, Schalock M. Autism Parenting Stress Index: Initial psychometric evidence. *J Autism Dev Disord* 2012;42:566–574.

(31) Emser TS, Mazzucchelli TG, Christiansen H, et al. Child Adjustment and Parent Efficacy Scale-Developmental Disability (CAPES-DD): First psychometric evaluation of a new child and parenting assessment tool for children with a developmental disability. *Res Dev Disabil* 2016;53-54:158–177.

(32) NHS Health Scotland, University of Warwick and University of Edinburgh. Short Warwick Edinburgh Mental Well-Being Scale (SWEMWBS). 2008. https://warwick.ac.uk/fac/sci/med/research/platform/wemwbs/development/swemwbs/

(33) Arnold DS, O'Leary SG, Wolff LS, et al. The Parenting Scale: A measure of dysfunctional parenting in discipline situations. *Psychol Assess* 1993;5:137–144.

(34) Rutter M, Bailey A, Lord C. Social Communication Questionnaire. Los Angeles, CA: Western Psychological Services 2003.

(35) Lord C, Rutter M, DiLavore PC, et al. Autism Diagnostic Observation Schedule (2nd ed.). Torrance, CA: Western Psychological Services 2012.

(36) Harrison P, Oakland T. Adaptive Behavior Assessment System (ABAS-3) (3rd ed.) Los Angeles, CA: Western Psychological Services 2015.

(37) Office of National Statistics. Measuring national well-being: Personal wellbeing in the UK, 2014 to 2015 (Statistical Bulletin). 2015. https://www.ons.gov.uk/peoplepopulationandcommunity/wellbeing/bulletins/measuringnationalwellbeing/2015-09-23

(38) EuroQol Group. EuroQol--A new facility for the measurement of health-related quality of life. *Health Policy* 1990;16:199–208.

(39) Beecham J, Knapp M. Costing psychiatric interventions. In: Thornicroft G, ed. Measuring Mental Health Needs (2nd ed). London: Royal College of Psychiatrists 2001:200–224.

For peer review only - http://bmjopen.bmj.com/site/about/guidelines.xhtml

(40) Curtis L, Burns A. Unit costs of health and social care 2017. Personal Social Service Research Unit, University of Kent, Canterbury 2017. https://www.pssru.ac.uk/project-pages/unit-costs/unit-costs-2017/

(41) Chan AW, Tetzlaff JM, Altman DG, et al. SPIRIT 2013 Statement: Defining standard protocol items for clinical trials. *Rev Panam Salud Publica* 2015;38:506–514.

For peer review only

For peer review only - http://bmjopen.bmj.com/site/about/guidelines.xhtml

Reporting checklist for protocol of a clinical trial.

Based on the SPIRIT guidelines.

Instructions to authors

Complete this checklist by entering the page numbers from your manuscript where readers will find each of the items listed below.

Your article may not currently address all the items on the checklist. Please modify your text to include the missing information. If you are certain that an item does not apply, please write "n/a" and provide a short explanation.

Upload your completed checklist as an extra file when you submit to a journal.

In your methods section, say that you used the SPIRIT reporting guidelines, and cite them as:

Chan A-W, Tetzlaff JM, Altman DG, Laupacis A, Gøtzsche PC, Krleža-Jerić K, Hróbjartsson A, Mann H, Dickersin K, Berlin J, Doré C, Parulekar W, Summerskill W, Groves T, Schulz K, Sox H, Rockhold FW, Rennie D, Moher D. SPIRIT 2013 Statement: Defining standard protocol items for clinical trials. Ann Intern Med. 2013;158(3):200-207

		Reporting Item	Page Number
Title	#1	Descriptive title identifying the study design, population, interventions, and, if applicable, trial acronym	1
Trial registration	#2a	Trial identifier and registry name. If not yet registered, name of intended registry	4

For peer review only

Trial registration: data set	#2b	All items from the World Health Organization Trial Registration Data Set	1-26
Protocol version	#3	Date and version identifier	24
Funding	#4	Sources and types of financial, material, and other support	25-26
Roles and responsibilities: contributorship	#5a	Names, affiliations, and roles of protocol contributors	25
Roles and responsibilities: sponsor contact information	#5b	Name and contact information for the trial sponsor	24
Roles and responsibilities: sponsor and funder	#5c	Role of study sponsor and funders, if any, in study design; collection, management, analysis, and interpretation of data; writing of the report; and the decision to submit the report for publication, including whether they will have ultimate authority over any of these activities	24-25
Roles and responsibilities: committees	#5d	Composition, roles, and responsibilities of the coordinating centre, steering committee, endpoint adjudication committee, data management team, and other individuals or groups overseeing the trial, if applicable (see Item 21a for data monitoring committee)	19, 22, 24

For peer review only

Background and rationale	#6a	Description of research question and justification for undertaking the trial, including summary of relevant studies (published and unpublished) examining benefits and harms for each intervention	5-7
Background and rationale: choice of comparators	#6b	Explanation for choice of comparators	5-6
Objectives	#7	Specific objectives or hypotheses	6-7
Trial design	#8	Description of trial design including type of trial (eg, parallel group, crossover, factorial, single group), allocation ratio, and framework (eg, superiority, equivalence, non-inferiority, exploratory)	9
Study setting	#9	Description of study settings (eg, community clinic, academic hospital) and list of countries where data will be collected. Reference to where list of study sites can be obtained	11, 17-18
Eligibility criteria	#10	Inclusion and exclusion criteria for participants. If applicable, eligibility criteria for study centres and individuals who will perform the interventions (eg, surgeons, psychotherapists)	9-10
Interventions: description	#11a	Interventions for each group with sufficient detail to allow replication, including how and when they will be administered	10-12

For peer review only

Interventions: modifications	#11b	Criteria for discontinuing or modifying allocated interventions for a given trial participant (eg, drug dose change in response to harms, participant request, or improving / worsening disease)	N/A
Interventions: adherance	#11c	Strategies to improve adherence to intervention protocols, and any procedures for monitoring adherence (eg, drug tablet return; laboratory tests)	12
Interventions: concomitant care	#11d	Relevant concomitant care and interventions that are permitted or prohibited during the trial	N/A
Outcomes	#12	Primary, secondary, and other outcomes, including the specific measurement variable (eg, systolic blood pressure), analysis metric (eg, change from baseline, final value, time to event), method of aggregation (eg, median, proportion), and time point for each outcome. Explanation of the clinical relevance of chosen efficacy and harm outcomes is strongly recommended	12-17
Participant timeline	#13	Time schedule of enrolment, interventions (including any run-ins and washouts), assessments, and visits for participants. A schematic diagram is highly recommended (see Figure)	17-18
Sample size	#14	Estimated number of participants needed to achieve study objectives and how it was determined, including clinical and statistical assumptions supporting any sample size calculations	12

For peer review only

Recruitment	#15	Strategies for achieving adequate participant enrolment to reach target sample size	17-18
Allocation: sequence generation	#16a	Method of generating the allocation sequence (eg, computer-generated random numbers), and list of any factors for stratification. To reduce predictability of a random sequence, details of any planned restriction (eg, blocking) should be provided in a separate document that is unavailable to those who enrol participants or assign interventions	9
Allocation concealment mechanism	#16b	Mechanism of implementing the allocation sequence (eg, central telephone; sequentially numbered, opaque, sealed envelopes), describing any steps to conceal the sequence until interventions are assigned	9, 18
Allocation: implementation	#16c	Who will generate the allocation sequence, who will enrol participants, and who will assign participants to interventions	9, 18
Blinding (masking)	#17a	Who will be blinded after assignment to interventions (eg, trial participants, care providers, outcome assessors, data analysts), and how	9, 18
Blinding (masking): emergency unblinding	#17b	If blinded, circumstances under which unblinding is permissible, and procedure for revealing a participant's allocated intervention during the trial	N/A

For peer review only

Data collection plan	#18a	Plans for assessment and collection of outcome, baseline, and other trial data, including any related processes to promote data quality (eg, duplicate measurements, training of assessors) and a description of study instruments (eg, questionnaires, laboratory tests) along with their reliability and validity, if known. Reference to where data collection forms can be found, if not in the protocol	12-18
Data collection plan: retention	#18b	Plans to promote participant retention and complete follow-up, including list of any outcome data to be collected for participants who discontinue or deviate from intervention protocols	18
Data management	#19	Plans for data entry, coding, security, and storage, including any related processes to promote data quality (eg, double data entry; range checks for data values). Reference to where details of data management procedures can be found, if not in the protocol	18-19
Statistics: outcomes	#20a	Statistical methods for analysing primary and secondary outcomes. Reference to where other details of the statistical analysis plan can be found, if not in the protocol	19-21
Statistics: additional analyses	#20b	Methods for any additional analyses (eg, subgroup and adjusted analyses)	N/A

For peer review only

Statistics: analysis population and missing data	#20c	Definition of analysis population relating to protocol non-adherence (eg, as randomised analysis), and any statistical methods to handle missing data (eg, multiple imputation)	19-20
Data monitoring: formal committee	#21a	Composition of data monitoring committee (DMC); summary of its role and reporting structure; statement of whether it is independent from the sponsor and competing interests; and reference to where further details about its charter can be found, if not in the protocol. Alternatively, an explanation of why a DMC is not needed	19, 22, 24
Data monitoring: interim analysis	#21b	Description of any interim analyses and stopping guidelines, including who will have access to these interim results and make the final decision to terminate the trial	N/A
Harms	#22	Plans for collecting, assessing, reporting, and managing solicited and spontaneously reported adverse events and other unintended effects of trial interventions or trial conduct	22
Auditing	#23	Frequency and procedures for auditing trial conduct, if any, and whether the process will be independent from investigators and the sponsor	N/A
Research ethics approval	#24	Plans for seeking research ethics committee / institutional review board (REC / IRB) approval	22

For peer review only

Protocol amendments	#25	Plans for communicating important protocol modifications (eg, changes to eligibility criteria, outcomes, analyses) to relevant parties (eg, investigators, REC / IRBs, trial participants, trial registries, journals, regulators)	24
Consent or assent	#26a	Who will obtain informed consent or assent from potential trial participants or authorised surrogates, and how (see Item 32)	22
Consent or assent: ancillary studies	#26b	Additional consent provisions for collection and use of participant data and biological specimens in ancillary studies, if applicable	N/A
Confidentiality	#27	How personal information about potential and enrolled participants will be collected, shared, and maintained in order to protect confidentiality before, during, and after the trial	19
Declaration of interests	#28	Financial and other competing interests for principal investigators for the overall trial and each study site	26
Data access	#29	Statement of who will have access to the final trial dataset, and disclosure of contractual agreements that limit such access for investigators	19
Ancillary and post trial care	#30	Provisions, if any, for ancillary and post-trial care, and for compensation to those who suffer harm from trial participation	N/A

For peer review only

Dissemination policy: trial results	#31a	Plans for investigators and sponsor to communicate trial results to participants, healthcare professionals, the public, and other relevant groups (eg, via publication, reporting in results databases, or other data sharing arrangements), including any publication restrictions	22-23
Dissemination policy: authorship	#31b	Authorship eligibility guidelines and any intended use of professional writers	N/A
Dissemination policy: reproducible research	#31c	Plans, if any, for granting public access to the full protocol, participant-level dataset, and statistical code	N/A
Informed consent materials	#32	Model consent form and other related documentation given to participants and authorised surrogates	On request from study team
Biological specimens	#33	Plans for collection, laboratory evaluation, and storage of biological specimens for genetic or molecular analysis in the current trial and for future use in ancillary studies, if applicable	N/A

The SPIRIT checklist is distributed under the terms of the Creative Commons Attribution License CC-BY-ND 3.0. This checklist can be completed online using https://www.goodreports.org/, a tool made by the EQUATOR Network in collaboration with Penelope.ai

For peer review only
