# Supplementary material for: A novel group parenting intervention to reduce emotional and behavioural difficulties in young autistic children: protocol for the Autism Spectrum Treatment and Resilience pilot randomised controlled trial
Source: BMJ Open. 2019 Jun 27;9(6):e029959. doi: 10.1136/bmjopen-2019-029959 (PMC6597639; doi:10.1136/bmjopen-2019-029959)
Supplement: Reviewer comments [file bmjopen-2019-029959.reviewer_comments.pdf]

PEER REVIEW HISTORY

BMJ Open publishes all reviews undertaken for accepted manuscripts. Reviewers are asked to complete a checklist review form (**http://bmjopen.bmj.com/site/about/resources/checklist.pdf**) and are provided with free text boxes to elaborate on their assessment. These free text comments are reproduced below.

ARTICLE DETAILS

**TITLE (PROVISIONAL)**	A novel group parenting intervention to reduce emotional and behavioural difficulties in young autistic children: Protocol for the Autism Spectrum Treatment and Resilience (ASTAR) pilot randomised controlled trial.
**AUTHORS**	Palmer, Melanie; Tarver, Joanne; Paris Perez, Juan; Cawthorne, Thomas; Romeo, Renee; Stringer, Dominic; Hallett, Victoria; Mueller, Joanne; Breese, Lauren; Hollett, Megan; Beresford, Bryony; Knapp, Martin; Slonims, Vicky; Pickles, Andrew; Simonoff, Emily; Scott, Stephen; Charman, Tony

This article was not externally reviewed at BMJ Open. Protocols that have been independently assessed prior to submission to BMJ Open are usually fast-tracked to publication on the grounds that further substantial changes will not be possible. This independent assessment will usually be external, independent review for both a substantial grant award from a non-commercial or government funder and ethics approval.
